# Coordinated transcriptional regulation of the carotenoid biosynthesis contributes to fruit lycopene content in high-lycopene tomato genotypes

**DOI:** 10.1093/hr/uhac084

**Published:** 2022-06-01

**Authors:** James R Duduit, Pawel Z Kosentka, Morgan A Miller, Barbara Blanco-Ulate, Marcello S Lenucci, Dilip R Panthee, Penelope Perkins-Veazie, Wusheng Liu

**Affiliations:** Department of Horticultural Science, North Carolina State University, Raleigh, NC, 27607, USA; Department of Horticultural Science, North Carolina State University, Raleigh, NC, 27607, USA; Department of Horticultural Science, North Carolina State University, Raleigh, NC, 27607, USA; Department of Plant Sciences, University of California, Davis, CA, 95616, USA; Dipartimento di Scienze e Tecnologie Biologiche ed Ambientali, Università del Salento (DiSTeBA), Via Prov.le Lecce-Monteroni, Lecce, 73100 Italy; Department of Horticultural Science, North Carolina State University, Mountain Horticultural Crops Research and Extension Center, Mills River, NC 28759, USA; Department of Horticultural Science, Plants for Human Health Institute, North Carolina State University, North Carolina Research Campus, Kannapolis, NC 28081, USA; Department of Horticultural Science, North Carolina State University, Raleigh, NC, 27607, USA

## Abstract

Lycopene content in tomato fruit is largely under genetic control and varies greatly among genotypes. Continued improvement of lycopene content in elite varieties with conventional breeding has become challenging, in part because little is known about the underlying molecular mechanisms in high-lycopene tomatoes (HLYs). We collected 42 HLYs with different genetic backgrounds worldwide. High-performance liquid chromatography (HPLC) analysis revealed lycopene contents differed among the positive control wild tomato *Solanum pimpinellifolium*, HLYs, the normal lycopene cultivar “Moneymaker”, and the non-lycopene cultivar NC 1Y at the pink and red ripe stages. Real-time RT-PCR analysis of expression of the 25 carotenoid biosynthesis pathway genes of each genotype showed a significantly higher expression in nine upstream genes (*GGPPS1*, *GGPPS2*, *GGPPS3*, *TPT1*, *SSU II*, *PSY2*, *ZDS*, *CrtISO* and *CrtISO-L1* but not the well-studied *PSY1*, *PDS* and *Z-ISO*) at the breaker and/or red ripe stages in HLYs compared to Moneymaker, indicating a higher metabolic flux flow into carotenoid biosynthesis pathway in HLYs. Further conversion of lycopene to carotenes may be prevented via the two downstream genes (*β-LCY2* and *ε-LCY*), which had low-abundance transcripts at either or both stages. Additionally, the significantly higher expression of four downstream genes (*BCH1*, *ZEP*, *VDE*, and *CYP97C11*) at either or both ripeness stages leads to significantly lower fruit lycopene content in HLYs than in the wild tomato. This is the first systematic investigation of the role of the complete pathway genes in regulating fruit lycopene biosynthesis across many HLYs, and enables tomato breeding and gene editing for increased fruit lycopene content.

## Introduction

Tomato (*Solanum lycopersicum* L.) is the most economically important specialty crop in the U.S., and the second only to potato in dietary consumption worldwide [1, 2]. The high consumption of tomato makes it a valuable dietary source of vitamin C, fibers, essential minerals, and carotenoids [3, 4]. Although other sources of dietary lycopene can be found in watermelon, GAC (*Momordica cochinchinensis*) fruit, pink grapefruit, and pink guava, the high consumption of tomatoes in the American diet accounts for about 85% of the lycopene consumed [5]. Lycopene is the primary carotenoid that gives tomato fruit a red color at the red ripe stage [6], and the red color of lycopene is one of the most significant decision factors in consumer acceptance of fresh market tomatoes and tomato processing products [7]. In tomato, lycopene begins to accumulate at the breaker stage of ripening and reaches maximal content at the red ripe stage. Fruit lycopene content is largely genetically controlled and varies among tomato genotypes [8]. Lycopene and other carotenoids, such as β-carotene and xanthophylls, act as photoprotective compounds in tomato fruit chromoplasts during ripening to protect cells from UV damage [9, 10]. The ability of lycopene to quench singlet oxygen also makes lycopene a powerful dietary antioxidant in human health, helping reduce the risk of diabetes, cardiovascular diseases, and cancer [3, 4].

Lycopene is an intermediate metabolite in the carotenoid biosynthesis pathway found in plastids. The carotenoid biosynthesis pathway uses isopentenyl diphosphate (IPP) from the methylerythritol 4-phosphate (MEP) pathway to make phytofluene and then lycopene, while lycopene acts as the substrate for other carotenoids including β-carotene, lutein and neoxanthin [11–13]. Most of these biosynthesis pathway genes are single copies and a few are multiple copies [13–15]. Starting from the breaker stage, expression of most genes upstream of lycopene biosynthesis is induced, while that of downstream genes of lycopene biosynthesis are naturally down-expressed [12, 16–21]. This leads to a significant increase in lycopene content in red ripe fruits of many tomato genotypes. For example, geranylgeranyl pyrophosphate synthase 2 (*GGPPS2*) and *GGPPS3* were upregulated across all fruit maturity stages in tomato genotype MP1 [22]. The phytoene synthase 1 (*PSY1*), phytoene desaturase (*PDS*), and ζ-carotene desaturase (*ZDS*) genes have been shown to be highly expressed at the breaker stage and decreased at the red ripe stage in the varieties Ailsa Craig, Moneymaker, Arka Ahuti, IIHR-249-1, IIHR-2866, VF36, and Red Setter [12, 16, 18, 20, 23–25]. *PSY2* became downregulated after the fruit entered into the breaker stage in Ailsa Craig [23, 24], but was slightly upregulated at red ripe stage in Red Setter [20]. In Moneymaker and Ailsa Craig, ζ-carotene isomerase (*Z-ISO*) had high expression at the breaker stage that lowered at the red ripe stage, while *ZDS*, carotenoid isomerase (*CrtISO*), *CrtISO-like 1* (*CrtISO-L1*) and *CrtISO-L2* had high expression at both breaker and red ripe stages [16]. In contrast, the downstream genes *ɛ-*lycopene cyclase (*ε-LCY*), *β-LCY2*, *CYP97A29*, *CYP97C11*, *β-*carotene hydroxylase 1 (*BCH1*), *BCH2*, zeaxanthin epoxidase (*ZEP*), violaxanthin desaturase (*VDE*), and neoxanthin synthase (*NSY*) had low expression at the breaker and red ripe stages in Arka Ahuti [12] and Red Setter [20], and *β-LCY1* had low expression at the breaker and red ripe stages in VF36 and Red Setter [20, 25]. Even though there exists a general trend for up- and down-regulated expression of most genes upstream and downstream the lycopene biosynthesis pathway, most of these tomato genotypes do not accumulate high levels of fruit lycopene content except Ailsa Craig and Red Setter which contain 100.0 and 85.5 μg/g FW lycopene [26, 27], respectively. Therefore, there exists genetic factors at play that affect the difference in fruit lycopene content, as demonstrated in molecular breeding using genetic engineering and genome editing [27–30].

Following tomato’s original domestication in Latin America and Mesoamerica, conventional tomato breeding efforts have largely focused on agronomic traits such as fruit size, increased shelf-life, and disease resistance rather than on red fruit color pigmentation, whose content decreased as domestication progressed [31, 32]. Several spontaneous mutations in the carotenoid biosynthesis pathway (*yellow-flesh*, *tangerine*, *delta*, *old-gold*, *old-gold-crimson*, *beta*, and *hp-3*) have been identified, which impact tomato fruit lycopene content [33]. The *yellow-flesh* and *tangerine* mutants have loss-of-function mutations in *PSY1* [6,11,34] and *CrtISO* [6,17,34], respectively, while the *delta* and *beta* mutants overexpressed *ɛ-LCY* [19] and *β-LCY2* [15], respectively. Each of these four mutants caused decreased fruit lycopene content. Conversely, *old-gold*/*old-gold-crimson* and *high pigment* (*hp*)*-3* are mutations in the carotenoid enzymatic *β-LCY2* [15] and *ZEP* [35] genes, respectively, leading to increased lycopene content in tomato fruit. Moreover, the *hp-1* and *-2* mutants, which contain mutated *UV-damaged DNA binding protein 1* (*ddb1*) and *DETIOLATED1* (*det1*) genes, respectively, exhibited high tomato fruit lycopene content due to significantly increased chromoplast size and number [36, 37]. These *og/hp-3*, *hp-1* and *-2* mutations have been used in conventional breeding for the generation and release of high-lycopene tomato genotypes across a wide range of genetic backgrounds worldwide [10]. The fruit lycopene content in these tomato genotypes varies from 22.7 μg/g FW (genotype Fla. 47) [38] to 303.8 μg/g FW (genotype HI-3518) [39].

According to Carli et al. [40] and Foolad [41], most of the high-lycopene tomato genotypes suffer from adverse pleiotropic effects of the mutated genes, such as slow germination and seedling growth, high seedling mortality, low leaf coverage, brittle stems, low yield, low soluble solids content, high susceptibility to various plant pathogens, and premature defoliation. The sum of these negative effects prohibits the widespread commercial employment of these varieties. However, Ilahy et al. [10] found that mixing the *hp-1* or *hp-2* mutations with non-mutant backgrounds might decrease the negative effects of the *hp* mutations in some high-lycopene tomato genotypes such as the commercially grown HLY13 and HLY18. To the best of our knowledge, these high-lycopene genotypes had never been subject to transcriptional analysis of the carotenoid biosynthesis pathway genes, and little is known about the underlying mechanisms regulating fruit lycopene content in high-lycopene genotypes.

The objective of this study was to systematically investigate the expression patterns of all carotenoid biosynthesis genes at different ripening stages of tomato fruit, and to link the differential gene expression patterns to the fruit lycopene content. We obtained 42 potential high-lycopene tomato genotypes (HLYs hereafter) with distinct genetic backgrounds from various international tomato breeding programs and companies for this comparative analysis. We hypothesized that fruit lycopene content in HLYs is under genetic control at the transcriptional levels of the genes in the carotenoid biosynthesis pathway. High-performance liquid chromatography (HPLC) was used to determine the content of trans-lycopene (lycopene hereafter), phytofluene, and β-carotene, and real-time RT-PCR (qPCR) was used to determine the relative gene expression levels of all the 25 carotenoid biosynthetic pathway genes. Overall, this large-scale analysis of high-lycopene tomato genotypes enabled the identification of the key pathway genes affecting fruit lycopene content that could be targeted for improving color and carotenoid levels in tomato commercial varieties.

## Materials and methods

### Plant materials and growth conditions

Seeds of 42 HLYs were obtained globally (Table S1). The wild tomato relative *S. pimpinellifolium* L. (LA2093; the wild tomato hereafter) was used as the positive control for a high-lycopene accumulating line, Moneymaker was used as a conventional control that produces normal quantity of fruit lycopene, while NC 1Y [42] was used as the negative control to represent non-red tomato lines. The wild tomato produces fruit with a bright red color and contains many desirable traits that have been lost in domesticated tomatoes [43]. Moneymaker is a red tomato line and has wild-type alleles for *hp-2^j^*/*hp-2^j^*. NC 1Y has the *tangerine* mutation in the encoding region of *CrtISO* and produces a significant amount of prolycopene at the expense of lycopene synthesis [17, 44].

All seeds were germinated in flat trays and grown in 3-G pots at 22–27°C in a greenhouse located at the Plants for Human Health in Kannapolis, NC from September 2018 to February 2019. Natural light was supplemented with Greenpower LED toplighting units (Phillips; Amsterdam, Netherlands) which provided an extra six hours of light per day with light intensity of 520 μmol/s. Three pots, each containing two plants per genotype, were placed in a randomized complete block design in the greenhouse and fertilized as needed.

Tomato fruits were collected from unpruned clusters in each replicate of each genotype at four fruit ripeness stages based on the USDA Visual Aid TM-L-1 (1975): 1) breaker (the beginning of yellow-orange in color covering <10%), 2) orange (orange in color covering 30–60%), 3) pink (pink to red in color covering 60–90%), and 4) red ripe (red in color covering 90–100%). Pericarp tissues, roughly 4 × 4 cm, were excised from each of the collected fruit samples using a clean scalpel. Half of the collected pericarp tissues (including fruit skin and pericarp tissues) was flash frozen using liquid nitrogen, ground to fine powder using a sterile mortar and pestle, and stored at −80°C for RNA extraction. The other half of the collected pericarp tissues was not ground but immediately placed in a −80°C freezer for carotenoid quantification using HPLC.

### HPLC for quantification of fruit contents of lycopene, β-carotene, and phytofluene

Pericarp samples from the pink and red ripe stages of each genotype (stored at −80°C) were allowed to thaw at room temperature and then pureed with a genogrinder (SPEX; Metuchen, NJ, USA). The purees were assayed for total soluble solids content (SSC) and acid content using digital refractometers (i.e. Pocket Pal and D5 Acid meter; Atago USA; Bellevue, WA, USA). Total lycopene content was assayed using the method of Davis et al. [45] and an UltraLab Color Scan Pro (Hunter Associates Laboratory; Reston, VA, USA). Results were used to determine relative amounts of puree needed for carotenoid extraction for HPLC.

Carotenoids (i.e. trans-lycopene, β-carotene, and phytofluene) was extracted using hexane:ethanol:acetone at a ratio of 2:1:1 following the method of Fish et al. [46]. Specifically, purees of red (0.1 to 0.3 g) and pink stages (0.6 g) were added to individual 40 mL amber vials. This was followed by the addition of 5 mL of 95% ethanol, 1 min vortex, addition of 10 mL of HPLC-grade hexane, 20 sec vortex, addition of 5 mL of acetone followed by manual inversion of vials. Vials were sonicated for 20 min, and shaken by hand half-way through. The vials were then placed on a shaker at 200 rpm for 15 minutes. Following the addition of 4 mL of double distilled water, all vials were shaken well by hand, and then placed back on the shaker for another 5 minutes. Samples were allowed to sit for 15 minutes, and if layer separation did not occur, the vials were cooled at −20°C for 5 minutes until separation occurred. One mL sample was pipetted and rolled down the side of each vial to ensure all residues were dislodged and dissolved then filtered through a 0.2 μM PTFE filter into HPLC vials, packed with N_2_ and stored at −80°C until all samples had been prepared for HPLC analysis.

Extracts (40 μL) were injected onto a HPLC (Elite; Hitachi High Technologies; Dallas, TX, USA) equipped with a diode array detector (DAD) and carotenoid C_30_ 4.6 × 250-mm column (YMC America; Allentown, PA, USA), controlled temperature auto sampler, and column compartment (35°C). Carotenoids were detected at wavelengths of 345 and 470 nm. The mobile phase consisted of 0.05% triethylamine (TEA) with 50 mM ammonium acetate in methanol (A), 0.05% TEA in 2-propanol (B), and 0.05% TEA with 250 mg/L BHT in THF (C) with a constant flow rate of 1 mL/min using a step gradient of 0 min, 90% A, 10% B; 24 min, 54% A, 35% B, 11% C; 35 min, 30% A, 35% B, 35% C; and 43–58 min, 90% A, 10% B. Calibration curves were determined using external standards of trans-lycopene, β-carotene, and phytofluene (CaroteNature; Ostermundigen, Munsingen, Switzerland) to identify and quantify carotenoids in samples. The D-2000 software (Hitachi; Kokubunji, Tokoyo, Japan) was used as the system run controller.

### RNA extraction and cDNA synthesis

RNA was extracted from 100 mg of the frozen powder of each fruit sample and purified using the Total Plant RNA Kit (Sigma; Burlington, MS, USA) and the On-Column DNase I treatment (Sigma; Burlington, MS, USA). Concentration and purity of each RNA sample were confirmed via Nanodrop ND-1000 spectrophotometer (Thermo Fisher; Wilmington, DE, USA) and 1% agarose gel electrophoresis. RNA was used for cDNA synthesis only if A260/A280 ratio was within the 1.9–2.1 range (indicating lack of contaminates) and 2 distinct bands representing 28S and 18S rRNA were shown on agarose gel with minimal streaking (indicating lack of degradation). First strand cDNA was synthesized from 1 μg of purified RNA using the High Capacity cDNA Reverse Transcription Kit (Applied Biosystems; Foster City, CA, USA). The synthesized cDNA was stored at −80°C.

### Sequence analysis and primer design

The carotenoid biosynthesis pathway genes plus two reference (internal control) genes *Expressed* (accession number: *Solyc07g025390.2.1*) and *Clathrin Adaptor Complex* (*CAC*, accession number: *Solyc08g006960.2.1*) [47, 48] were included in the present study (Tables S2; S3). The selection of the two reference genes was based on their high reference stability and function together when assaying tomato fruit tissues [47, 48]. The protein sequence of each gene was obtained from Genbank and used as the query sequence to search against the tomato whole genome sequences in the Phytozome database (v12.1; https://phytozome-next.jgi.doe.gov/) using TBLASTN. The deduced protein sequences of all the returned sequences of each gene were used for protein sequence alignment using ClustalX 2.0 (http://www.clustal.org/). The sequences that obviously lacked sequence homology were removed, and the remaining homologous sequences of each gene were used for cDNA sequence alignment by using their cDNA sequences (Figure S1). Primer design was conducted as described in our newly published stepwise qPCR optimization method [49]. Specifically, the single nucleotide polymorphisms (SNPs) present in the cDNA alignment of each gene with its homologs were used for gene-specific primer design for each gene. Two forward and two reverse primers of 20–23 bp in length with 45–55% GC content were designed next to each other for each gene with the SNPs being located at the last nucleotide position (or more positions including the last one) at the 3′-end of each primer. These primers formed four primer pairs for each of the 25 genes plus the 2 reference genes with the PCR amplicons being 85–125 bp in length (including the length of the two primers) if possible (Tables S2; S3).

### Optimization of qPCR conditions

Stepwise optimization of qPCR conditions was conducted as described in Zhao et al. [49] with the cDNA from the red ripe stage of the randomly chosen genotype, Amai, so that R^2^ ≥ 0.99 and Efficiency (E) = 100 ± 5% could be achieved for the standard cDNA concentration curve with a logarithmic scale for the best primer pair for each gene (Table S3). This served as the prerequisite for using the 2^−ΔCt^ and 2^−ΔΔCt^ methods [50–52] for data analysis.

### qPCR

The relative transcript abundance of each gene at the breaker and red ripe stages of each genotype was quantified by qPCR using the optimized qPCR conditions for the best primer pair of each gene (Tables S2; S3) and *Expressed* and *CAC* as the two reference genes. qPCR was performed with three technical replicates on clear plastic 96-well plates with optical film (Bio-Rad; Hercules, CA, USA) on a CFX96 Touch Real-Time PCR Detection System (Bio-Rad; Hercules, CA, USA). Each 10 μL reaction volume consisted of 5 μL SYBR Master Mix (#4344463, Thermo Fisher; Waltham, MA, USA), 0.25–0.35 μL of forward and reverse primers (10 μM), 1 μL of diluted cDNA of the red ripe fruit of Amai, and nuclease-free water. The PCR product was amplified at an initial 95°C for 2 minutes, then 39 cycles of 95°C for 5 seconds and 59°C for 30 seconds. Biological replicates were performed in triplicate and their Ct values were averaged.

Data analysis for relative expression level of each gene was conducted with the 2^-ΔCt^ method where the mean Ct value of each gene of interest (GOI) was subtracted from the geometric mean of the two reference genes: ΔCt = Ct_GOI_ – Ct_Reference Gene_ [50, 52]. Fold changes in relative gene expression of each GOI in each genotype was calculated using the 2^-ΔΔCt^ method with that in Moneymaker (for upstream genes) and the wild tomato (for downstream genes): ΔΔCt = ΔCt – ΔCt_Control_ [50, 52].

### Statistical analysis

A correlation coefficient analysis was performed using simple linear regression to compare carotenoid quantity and entire pathway gene expression to identify gene(s) that contribute to higher fruit lycopene content. Statistical analyses of qPCR and HPLC were performed via a two-tailed student’s *t*-test with two-sample unequal variance.

## Results

### Quantification of fruit contents of lycopene, β-carotene, and phytofluene at the pink and red ripe fruit ripening stages in HLYs using HPLC analysis

**Figure 1:**
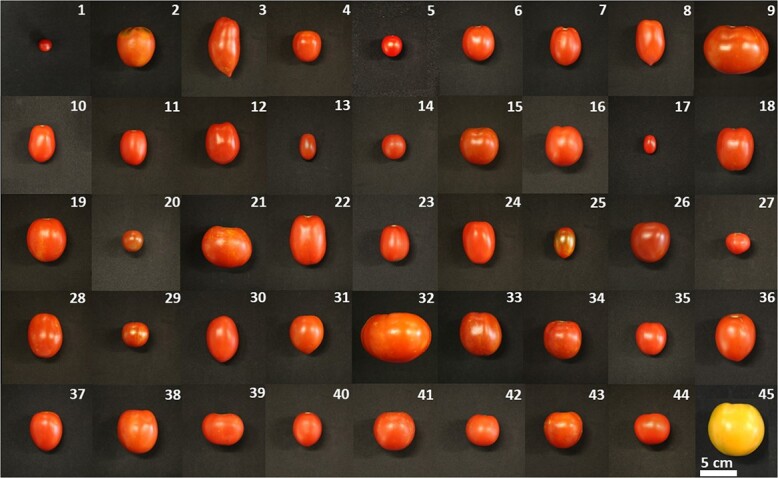
Representative tomato fruit images of the 42 HLYs at the red ripe fruit ripening stage (refer to Table S1 for corresponding genotype names). The wild tomato *S. pimpinellifolium* L. (LA 2093; #1) was used as the positive control, Moneymaker (#44) was used a conventional low-lycopene control, and NC 1Y (#45) was used as the negative control.

Tomato fruits were harvested from the 42 HLYs, the positive control wild tomato, the low-lycopene accumulating control Moneymaker, and the negative control NC 1Y at the pink and red ripe stages, and fruit contents of lycopene, β-carotene, and phytofluene were quantified in pericarp tissues using HPLC. Fruits harvested from each genotype at the breaker and orange stages were excluded from our HPLC analysis due to the large variability in the fruit content of each of the three carotenoids from sample to sample for each genotype. As shown in Figure 1, these genotypes showed various fruit sizes and shapes at the red ripe stage with highly similar bright red color as the wild tomato and Moneymaker but very different from NC 1Y, which showed an orange color due to the *tangerine* mutation in the *CrtISO* gene.

**Figure 2:**
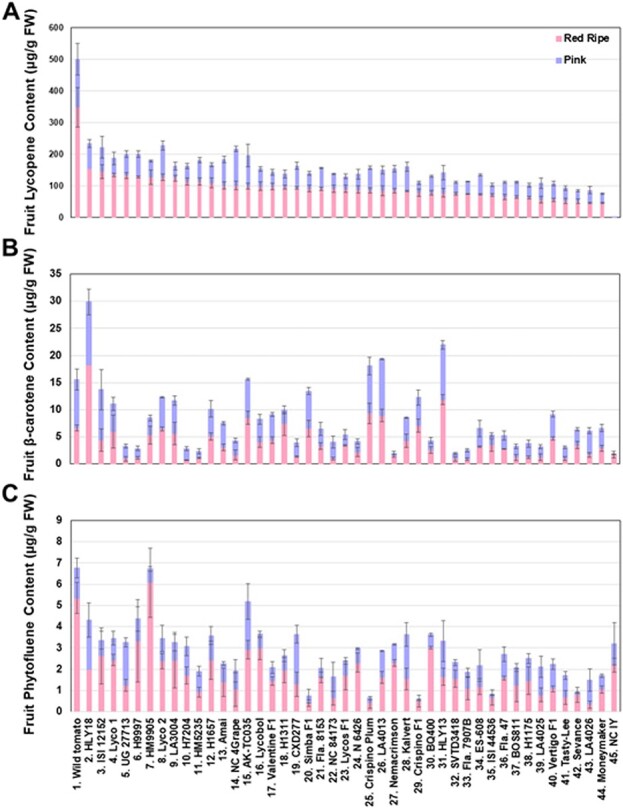
Fruit contents of lycopene (A), β-carotene (B), and phytofluene (C) in the 42 HLYs at the pink and red ripe stages of fruit ripeness. The wild tomato *S. pimpinellifolium* L. (LA 2093) was used as the positive control, Moneymaker was used a conventional low-lycopene control, and NC 1Y was used as the negative control. All of the cultivars were grown under the same greenhouse conditions at the same time, and HPLC was used to quantify fruit lycopene, β-carotene and phytofluene contents in pericarp tissues. FW, fresh weight.

At the red ripe stage, the highest fruit lycopene content (348.8 μg/g FW) was found in the wild tomato, followed by 153.6 and 145.2 μg/g FW for HLY18 and ISI12152, respectively (Figure 2A). The lowest lycopene content was 46.5 μg/g FW in LA4026 and this value was comparable to the 46.2 μg/g FW found in Moneymaker. As expected, the negative control NC 1Y contained 0.2 and 0.0 μg/g FW lycopene at the pink and red ripe stages, respectively. Overall, we found that the fruit lycopene content in 19 out of the 42 HLYs at the red ripe stage was at least two times higher than that of Moneymaker.

At the pink stage, fruit lycopene contents ranged from 31.8 μg/g FW in Crispino F1 to 152.2 μg/g FW in the wild tomato (Figure 2A). The average increase in lycopene contents from pink to red ripe stages was 1.6-fold. All of the genotypes exhibited a steady increase in fruit lycopene content from pink to red ripe stages except NC 4Grape and NC 1Y. The average fruit lycopene contents in HLYs at the pink and red ripe stages were 57.7 and 92.4 μg/g FW, respectively, which were significantly higher than that in Moneymaker (28.7 and 46.2 μg/g FW; *p* < 0.05) and NC 1Y (*p* < 0.05) (Figure S1).

Unlike lycopene, fruit β-carotene contents exhibited a general decrease in most genotypes from pink to red ripe stages (Figure 2B). At the pink stage, fruit β-carotene contents ranged from 0.5 μg/g FW in Nemacrimson to 11.6 μg/g FW in HLY18. The average fruit β-carotene content in HLYs was 4.1 μg/g FW, which was not significantly different from that in the wild tomato (9.0 μg/g FW) and Moneymaker (3.7 μg/g FW) but was significantly higher than that in NC 1Y (0.1 μg/g FW; *p* < 0.05) (Figure S1). At the red ripe stage, fruit β-carotene contents varied from 0.6 μg/g FW in H7204 to 18.3 μg/g FW in HLY18. The average β-carotene content in HLYs at the red ripe stage (4.1 μg/g FW) was insignificantly different from that in Moneymaker (2.9 μg/g FW) and NC 1Y (1.7 μg/g FW), but significantly lower than that in the wild tomato (6.6 μg/g FW; *p* < 0.05) (Figure S1). The fruit β-carotene contents were about 8–10 times less than the lycopene contents in almost every genotype at the red ripe stage. Interestingly, HLY18 produced the highest fruit β-carotene content and the second highest lycopene content (Figure 2B).

Similar to fruit lycopene contents, we observed a gradual increase in fruit phytofluene contents in most genotypes across fruit ripening process (Figure 2C). Fruit phytofluene contents ranged from 0.1 μg/g FW in Crispino F1 to 2.3 μg/g FW in HLY18, AK-TC035, and CXD277 at the pink stage, and from 0.3 μg/g FW in LA4026 to 6.1 μg/g FW in HM9905 at the red ripe stage. The average contents in HLYs were significantly higher than that in Moneymaker at the pink (1.0 vs 0.6 μg/g FW; *p* < 0.05) and red ripe (1.7 vs 1.0 μg/g FW; *p* < 0.05) stages but significantly lower than that in the wild tomato at the red ripe stage (5.4 μg/g FW; *p* < 0.05) (Figure S1). The average increase in phytofluene from pink to red ripe stages was 1.7-fold in HLYs. At the red ripe stage, we noticed that fruit phytofluene contents were half or less than half of β-carotene contents in almost every genotype, and the two highest phytofluene contents came from HM9905 and the wild tomato, which produced the 7th highest and the highest lycopene contents, respectively (Figure 2C).

**Figure 3:**
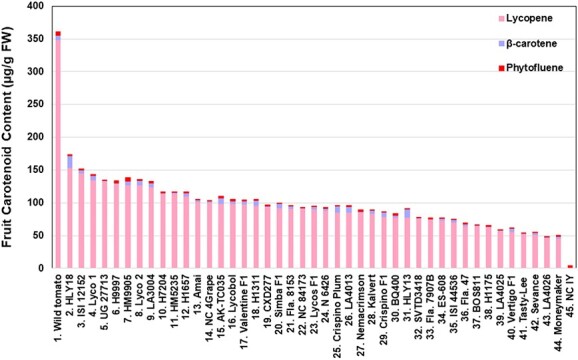
Total fruit carotenoid (lycopene, β-carotene and phytofluene) contents in the fruits of the 42 HLYs at the red ripe fruit developmental stages. The wild tomato *S. pimpinellifolium* L. (LA 2093) was used as the positive control, Moneymaker was used a conventional low-lycopene control, and NC 1Y was used as the negative control. All of the cultivars were grown under the same greenhouse conditions at the same time, and HPLC was used to quantify fruit lycopene, β-carotene and phytofluene contents in pericarp tissues. FW, fresh weight.

When all the three carotenoids at the red ripe stage were combined for each genotype, we found that >99.9% of carotenoids at the red ripe stage of each genotype came from lycopene except NC 1Y which does not produce lycopene (Figure 3).

### Optimization of qPCR conditions in genotype Amai

Primer design started with the BLASTN search against the tomato whole genome sequences using individual carotenoid biosynthesis pathway genes as the query sequences, which returned a total of 25 carotenoid biosynthesis pathway genes with high protein sequence homology (Figure S2). Phylogenetic analysis of the protein sequences of these 25 genes together with the reference genes *Expressed* and *CAC* grouped them into 12 groups (Figure S2). Based on the cDNA sequence alignment of the genes within each group, the SNPs identified among the genes within each group were used for sequence-specific primer design for qPCR of each gene (Table S2; Figures S3-S12) according to our newly published stepwise qPCR optimization method [49]. Four sequence-specific primer pairs of 20–22 bp in length with 45–55% GC content were designed for optimization of qPCR conditions for each of the 27 genes (Table S2; Figures S3-S12). The PCR amplicons were 80–117 bp in length (including the length of the two primers) for all of the primer pairs of each gene with seven exceptions which had PCR amplicons of 77–79, 121 or 128 bp in length (Table S3).

We randomly chose the red ripe pericarp tissues from the genotype Amai to optimize the qPCR conditions as described in Zhao et al. [49]. Optimization of the qPCR conditions for each primer pair of each gene was conducted sequentially by optimizing primer annealing temperature and primer concentration and identifying the optimal template cDNA concentration range and the best primer pair for each gene [49]. Using a 1:10 diluted pericarp cDNA from the red ripe fruit as the templates and a 350 mM primer concentration for each primer in each reaction, we conducted gradient qPCR at 52.0, 54.1, 56.8, 59.0, and 60.2°C to identify the optimal annealing temperature for each primer pair of each gene. The Ct values were between 20.1 and 29.9 for most primer pairs. For the primer pairs whose Ct values were larger than 30.0, we repeated the gradient qPCR by using a 1:5 diluted red ripe fruit cDNA as the templates. The qPCR reactions at 56.8 or 59.0°C had the lowest Ct values for each primer pair for the 25 pathway genes, while that at 56.8 and 59.0°C had the lowest Ct values for most primer pairs for the 2 reference genes (Table S3). Thus, the annealing temperature at 56.8 and/or 59.0°C provided an optimal temperature for each primer pair for each gene. The only exceptions came from two primer pairs for *GGPPS1*, *PSY3*, *BCH2*, and *ZEP*, which failed to amplify their respective genes (Table S3).

Using the same red ripe Amai pericarp cDNA (1:10 or 1:5 diluted) as the templates and 56.8 and/or 59.0°C as the optimal annealing temperatures, we used primer concentrations of 200, 250, 300, 350, and 400 mM to determine the optimal primer concentration for each primer pair. We found primer concentrations of 250, 300 or 350 mM per reaction had the lowest Ct values for most primer pairs (Table S3). Thus, the primer concentration with the lowest Ct value was chosen as the optimal primer concentration for that primer pair (Table S3).

Using the optimal annealing temperature at 56.8 and/or 59.0°C and the optimal primer concentration for each primer pair as shown in Table S3, we used serial dilutions of the same red ripe fruit cDNA (1:5, 1:10, 1:20, 1:40, 1:80, and 1:160 dilutions) as the templates to qPCR amplify each gene. We obtained the standard concentration curve with a logarithmic scale for each primer pair and factored the PCR efficiency of each primer pair into an equation (Figure 4). We found that most R^2^ were between 0.9800 and 1.0000, and most efficiencies were between 100 ± 5% under the conditions of the optimal annealing temperature and primer concentration and various cDNA serial dilutions (Table S3). The best primer pair for each gene gave rise to the best R^2^ (0.9912–1.0000) and efficiencies (97.0–105.0%) while using the optimal annealing temperature at 59.0°C (Figure 4; Table S3). The optimal qPCR template cDNA concentration ranges varied from gene to gene, but all of them included the 1:20 diluted red ripe pericarp cDNA, i.e. Log (cDNA in ng/reaction) = 0.39794 (Figure 4).

**Figure 4:**
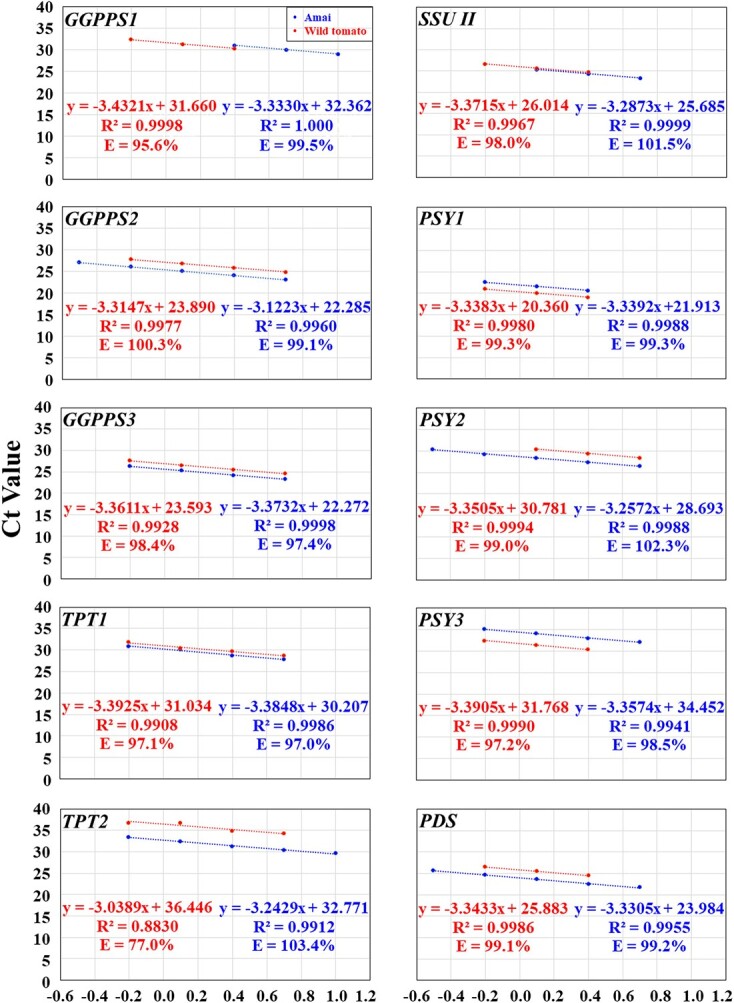
The plot of the averaged Ct values from three technical replicates against the Log (cDNA in ng/reaction) for optimizing qPCR conditions for the best primer pair of each of the 25 carotenoid biosynthesis genes plus 2 reference genes in the pericarp tissues of the genotype Amai and the wild tomato *S. pimpinellifolium* L. (LA 2093) at the red ripe stage. The PCR efficiency (E; %) for each primer pair was calculated as E = (10^–1/A^ − 1) × 100 in the equation y = Ax + B for each gene. The cDNA concentration in 1:5, 1:10, 1:20, 1:40, 1:80, and 1:160 dilutions was 10, 5, 2.5, 1.25, 0.625, 0.3125 ng/μl, respectively, while the Log (cDNA in ng/reaction) for the 1:5, 1:10, 1:20, 1:40, 1:80, and 1:160 dilutions were 1.0000, 0.69897, 0.39794, 0.09691, − 0.20412, and − 0.50515, respectively. The data from the lowest (or highest) one (or two or three) cDNA concentration might have been omitted in order to obtain R^2^ ≥ 0.99 and E = 100 ± 5% for the data from the remaining four (or three) consecutive cDNA concentrations for the best primer pair for each gene. This served as the prerequisite for using the 2^−ΔCt^ and 2^−ΔΔCt^ methods for data analysis.

**Figure 4:**
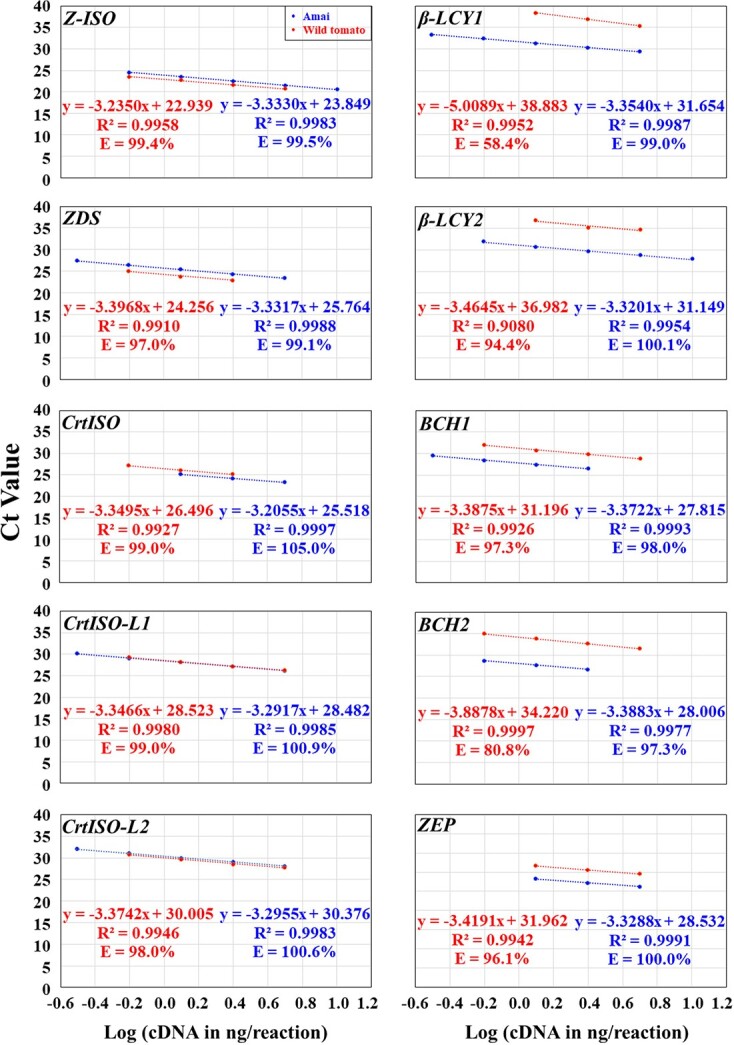
Continued

**Figure 4:**
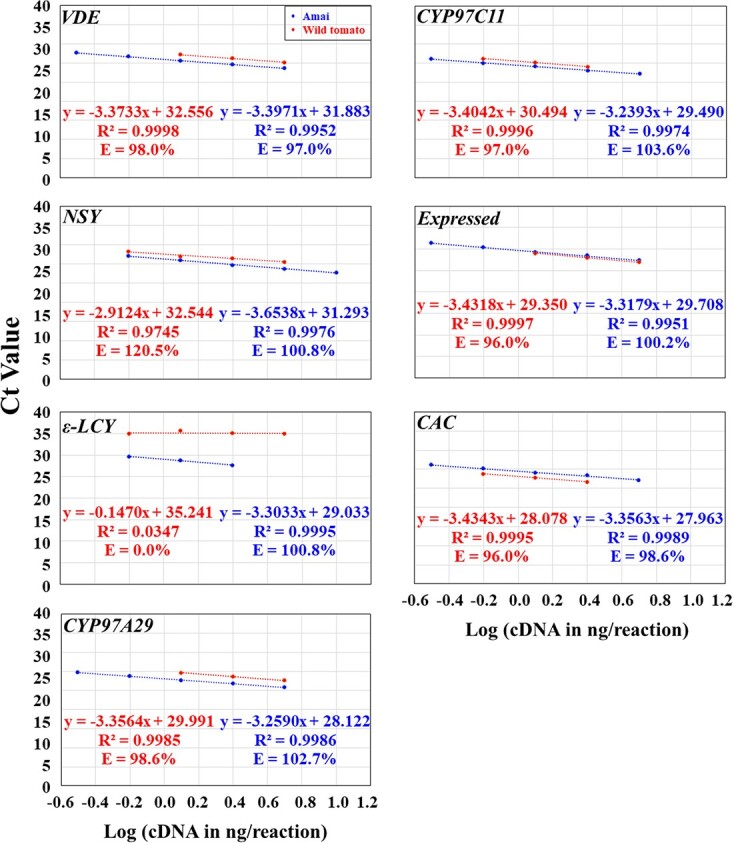
Continued

### Test of the optimized qPCR conditions of the best primer pair of each gene in the wild tomato

Since the domesticated tomato genepool contains <5% of the genetic variation found in wild tomatoes [53], and consequently few nucleotide polymorphisms [54–58], we tested whether the optimized qPCR conditions for the best primer pair of each gene in Amai would be suitable for qPCR analysis of each gene in the wild tomato. We applied the optimized primer annealing temperature and concentration to identify the optimal template cDNA concentration range for each gene in the wild tomato by using a 1:10 diluted pericarp cDNA from the red ripe fruit as the templates. As shown in Figure 4, the best primer pair of each gene had 0.9908 ≤ R^2^ ≤ 0.9998 and 95.6% ≤ E ≤ 100.3% under the optimized conditions and various cDNA serial dilutions. The only exceptions came from *TPT2* (R^2^ = 0.8830; E = 77.0%), *β-LCY1* (E = 58.4%), *β-LCY2* (R^2^ = 0.9080; E = 94.4%), *BCH2* (E = 80.8%), *NSY* (R^2^ = 0.9745; E = 120.5%), and *ε-LCY* (R^2^ = 0.0347; E = 0.0%); these genes are either silenced or minimally expressed in the wild tomato (see below). The optimal qPCR template cDNA concentration ranges also varied from gene to gene, but all of them included the 1:20 and 1:40 diluted red ripe pericarp cDNA, i.e. Log (cDNA in ng/reaction) = 0.39794 and 0.09691, respectively (Figure 4).

Therefore, the optimized qPCR conditions were suitable for qPCR analysis of most of the 25 genes plus 2 internal controls in the wild tomato and used for further qPCR analysis in the present study, and the 1:20 diluted pericarp cDNA was chosen to be used as the templates for further qPCR analysis of each gene in each genotype at 59.0°C.

### Relative expression levels of the upstream genes in the fruits of HLYs at the breaker and red ripe stages

To understand whether and how fruit lycopene contents are transcriptionally regulated in HLYs, we used qPCR to analyze the relative expression levels of the complete carotenoid biosynthesis pathway genes in the pericarp tissues at the breaker and red ripe stages of each genotype. We grouped the 42 HLYs into one group by stage of ripeness and compared relative expression levels of the remaining upstream genes with the high-lycopene control (the wild tomato) and low-lycopene control (Moneymaker) fruit. We also grouped the 42 HLYs into 4 and 5 subgroups based on the fruit lycopene content at the red ripe stage (such as >150, 100–150, 50–100, <50 μg/g FW for 4 subgroups). As results were very similar statistically, we are displaying results from the one group statistical assay.

**Figure 5:**
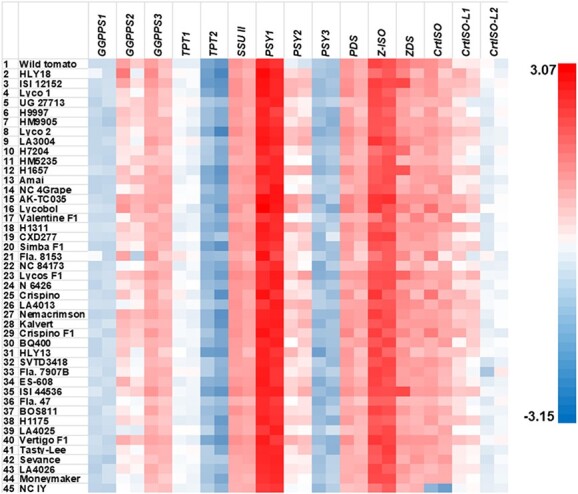
Heat map of relative expression levels of the fifteen upstream genes in the fruits of the 42 HLYs at the breaker and red ripe stages measured by qPCR. The relative expression of each gene was measured by qPCR, and relative quantification was performed using our newly published stepwise qPCR optimization method [49] with the tomato *Expressed* and *CAC* genes as the reference genes. The mean of the relative expression levels of the three biological replicates were log transformed. The wild tomato *S. pimpinellifolium* L. (LA2093) was used as the positive control genotype, Moneymaker was used a conventional low-lycopene control, and NC 1Y was used as the negative control. For each gene in each line, left and right boxes represent the breaker and red ripe stages, respectively.

Using *Expressed* and *CAC* as the two reference genes [47,48], the relative gene expression of all the pathway genes showed a general trend of strong expression of most upstream genes and weak expression of most downstream genes in all genotypes at both developmental stages in each genotype (Figure 5). Among the upstream genes in all genotypes, *PSY1*, *Z-ISO* and *ZDS* consistently had the highest, the second and third highest relative expression levels, respectively, followed by *SSU II*, *CrtISO PDS*, *GGPPS2*, *GGPPS3*, and *PSY2* (Table 1). All of these genes had relative expression levels larger than 1, indicating higher relative expression levels than the reference genes (Table 1). In contrast, *GGPPS1*, *TPT1*, *TPT2*, *PSY3* and *CrtISO-L2* had relative expression levels less than 1, showing lower relative expression than the reference genes (Table 1).

**Table 1:** **Comparison of the relative expression levels of the carotenoid biosynthesis pathway genes in the fruits of the 42 potential high-lycopene tomato genotypes (HLYs) at breaker and red ripe stages.** The wild tomato *S. pimpinellifolium* L. (LA2093) was used as the positive control, Moneymaker was used a conventional low-lycopene control, and NC 1Y was used as the negative control. The NC 1Y non-functional *crtiso* gene was used as the negative control gene

**Gene**	**Breaker Stage**			**Red Ripe Stage**		
	**Wild Tomato** ^**a**^	**HLYs** ^**b**^	**Moneymaker** ^**c**^	**Wild Tomato** ^**a**^	**HLYs** ^**b**^	**Moneymaker** ^**c**^
*Upstream genes*						
*GGPPS1*	0.13 ± 0.01	0.15 ± 0.12^***^	0.10 ± 0.02^*^	0.14 ± 0.03	0.13 ± 0.05^***^	0.17 ± 0.07
*GGPPS2*	11.86 ± 6.99	4.70 ± 7.11^***^	0.62 ± 0.22^***^	3.51 ± 0.38^**^	2.31 ± 2.14^***^	0.41 ± 0.10^***^
*GGPPS3*	12.44 ± 8.60	9.40 ± 5.27^***^	6.54 ± 1.25^*^	5.25 ± 0.54	6.13 ± 2.52^***^	3.30 ± 0.53^***^
*TPT1*	0.33 ± 0.15	0.62 ± 0.38^***^	0.84 ± 0.77	0.34 ± 0.06^**^	0.55 ± 0.27^***^	0.43 ± 0.04^**^
*TPT2*	0.01 ± 0.004^***^	0.03 ± 0.03	0.04 ± 0.04	0.004 ± 0.001^***^	0.01 ± 0.01	0.01 ± 0.01
*SSU II*	16.11 ± 1.71	13.94 ± 4.54^***^	7.89 ± 1.58^**^	9.84 ± 0.50	10.43 ± 3.57^***^	6.29 ± 0.18^***^
*PSY1*	379.19 ± 38.21^*^	476.9 ± 293.8^***^	283.1 ± 105.3	269.7 ± 83.6	383.5 ± 156.3^***^	367.6 ± 97.0
*PSY2*	0.39 ± 0.21^***^	3.47 ± 4.36^***^	1.31 ± 0.84^*^	0.19 ± 0.07^***^	3.57 ± 3.30^***^	0.82 ± 0.28^***^
*PSY3*	0.05 ± 0.03	0.04 ± 0.03	0.05 ± 0.04	0.04 ± 0.03	0.06 ± 0.07^***^	0.10 ± 0.02^*^
*PDS*	15.68 ± 7.03	13.25 ± 8.08^***^	8.60 ± 4.26	8.13 ± 0.90	7.84 ± 3.51^***^	6.29 ± 1.19
*Z-ISO*	170.5 ± 76.5	131.2 ± 89.7^***^	145.4 ± 33.3	116.8 ± 3.74	120.1 ± 59.9^***^	138.1 ± 27.3
*ZDS*	40.34 ± 27.02	25.17 ± 58.92^***^	10.05 ± 2.64^**^	20.80 ± 1.04^***^	11.14 ± 6.13^***^	5.62 ± 1.25^**^
*CrtISO*	18.38 ± 15.68	13.93 ± 14.31^***^	7.75 ± 2.94^*^	9.12 ± 1.75	8.66 ± 5.39^***^	8.25 ± 1.86
*CrtISO-L1*	1.32 ± 0.07^***^	3.44 ± 3.72^***^	1.30 ± 0.63^**^	1.49 ± 0.11^***^	2.32 ± 2.03^***^	0.82 ± 0.45^**^
*CrtISO-L2*	0.93 ± 1.09	0.39 ± 0.32^***^	0.31 ± 0.10	0.30 ± 0.07^***^	0.64 ± 0.32^***^	0.51 ± 0.14
*Downstream genes*						
*β-LCY1*	0.01 ± 0.01^**^	0.07 ± 0.10^**^	0.04 ± 0.06	0.004 ± 0.001^***^	0.08 ± 0.10^***^	0.02 ± 0.01^***^
*β-LCY2*	0.01 ± 0.01	0.02 ± 0.03	0.01 ± 0.01	0.002 ± 0.001^***^	0.01 ± 0.01	0.002 ± 0.001^***^
*BCH1*	0.38 ± 0.20^**^	1.12 ± 1.60^***^	0.54 ± 0.13^**^	0.16 ± 0.07^***^	1.51 ± 1.22^***^	1.68 ± 0.22
*BCH2*	0.02 ± 0.02^**^	0.07 ± 0.10^**^	0.02 ± 0.01^***^	0.01 ± 0.01^*^	0.04 ± 0.03^***^	0.02 ± 0.01^**^
*ZEP*	0.75 ± 0.38	1.18 ± 1.09^***^	0.70 ± 0.44	0.16 ± 0.05^***^	1.01 ± 0.76^***^	0.73 ± 0.37
*VDE*	0.12 ± 0.05^***^	2.51 ± 2.46^***^	0.80 ± 0.41^**^	0.04 ± 0.03^***^	1.66 ± 1.61^***^	0.33 ± 0.28^***^
*NSY*	0.10 ± 0.04	0.08 ± 0.07^***^	0.25 ± 0.14	0.06 ± 0.01	0.05 ± 0.05^***^	0.14 ± 0.04^*^
*ε-LCY*	0.01 ± 0.01^***^	0.08 ± 0.16^***^	0.05 ± 0.05	0.004 ± 0.003^*^	0.01 ± 0.02	0.04 ± 0.06
*CYP97A29*	0.52 ± 0.11	0.55 ± 0.40^***^	0.20 ± 0.01^***^	0.23 ± 0.05	0.32 ± 0.19^***^	0.13 ± 0.02^***^
*CYP97C11*	0.40 ± 0.21^***^	1.56 ± 1.31^***^	1.11 ± 0.46	0.21 ± 0.06^***^	0.80 ± 0.62^***^	0.54 ± 0.17

a Average ± standard deviation of the relative expression level of each gene in the wild tomato, and the statistical significance between the wild tomato and the average of HLYs.

b Average ± standard deviation of the relative expression level of each gene in HLYs, and the statisticalsignificance between the average of HLYs and the NC IY non-functional *crtiso* gene that was 0.0177 ± 0.0129 and 0.0058 ± 0.0033, respectively, at the breaker and red ripe stages.

c Average ± standard deviation of the relative expression level of each gene in Moneymaker, and the statistical significance between Moneymaker and the average of HLYs. ^*^ denotes *p*-value <0.05 to 0.01; ^**^ denotes *p*-value = 0.01 to 0.001; and ^***^ denotes *p*-value <0.001 using a two-tailed student’s *t*-test with two-sample unequal variance, i.e. significantly different expression from that in controls.

Since the *tangerine* mutation resulted in a non-functional *crtiso* in NC 1Y [17, 44], we compared the relative expression levels of these upstream genes in HLYs as a group with that of *crtiso* in NC 1Y at both stages (0.0177 ± 0.0129 and 0.0058 ± 0.0033, respectively). We found that most of these upstream genes at either stage as a group had significantly higher relative expression levels than that of *crtiso* in NC 1Y (Table S1). The only exception came from the relative expression levels of *TPT2* at both stages and *PSY3* at the breaker stage, which were insignificantly different from that of *crtiso* in NC 1Y (Table 1; Figures 5; S13). In addition, the relative expression level of *PSY3* at the red ripe stage was extremely low (0.06 ± 0.07; Table 1). Thus, expression of *TPT2* and *PSY3* at both stages of HLYs as a group were silenced or minimal. Similarly, *TPT2* and *PSY3* at both stages of the wild tomato were silenced or minimally expressed (Table 1).

When compared to Moneymaker, we found that *GGPPS1* and *CrtISO* at the breaker stage, *GGPPS2*, *GGPPS3*, *SSU II*, *PSY2*, *ZDS* and *CrtISO-L1* at both stages, and *TPT1* at the red ripe stage had significantly higher relative expression levels in HLYs as a group (Table 1; Figures 5; S13). Thus, higher relative expression of these nine upstream genes (i.e. *GGPPS1*, *GGPPS2*, *GGPPS3*, *TPT1*, *SSU II*, *PSY2*, *ZDS*, *CrtISO* and *CrtISO-L1*) at either or both stages contributed to higher fruit lycopene contents in HLYs than in Moneymaker. The fold changes of the relative expression levels of these genes in HLYs ranged from 1.44 in *GGPPS3* to 2.65 in *PSY2* and *CrtISO-L1* and from 1.66 in *SSU II* to 2.83 in *CrtISO-L1* at the red ripe stage when compared to Moneymaker. *GGPPS2* increased 7.58 folds at the breaker stage, and *PSY2* and *GGPPS2* increased 4.35 and 5.63 folds, respectively.

In comparison to wild tomato, *PSY1* at the breaker stage, *PSY2* and *CrtISO-L1* at both stages, and *CrtISO-L1* at the red ripe stage had significantly higher relative expression levels in HLYs as a group while *ZDS* at the red ripe stage had a significantly lower relative expression levels in HLYs (Table 1; Figures 5; S13). Therefore, the precise transcriptional regulation of these five genes contributed to the lower fruit lycopene contents in HLYs than in the wild tomato. It is worthwhile to point out that *CrtISO-L2* showed significantly higher relative expression levels in 7 out of the 42 HLYs at the red ripe stage than that at the breaker stage (Figure S13).

Surprisingly, *PSY1*, *PDS* and *Z-ISO* did not show significant difference in relative expression levels at both stages of HLYs as a group from that in Moneymaker or wild tomato (except *PSY1* at the breaker stage) even though they were highly expressed (Table 1). At the breaker stage, the relative expression levels of *PSY1* ranged from 221.84 in LA4013 to 1182.8 in Lycobol with an overall average of 477.80 in HLYs, *PDS* ranged from 5.27 in NC 84173 to 31.64 in Lycobol with an average of 13.26 in HLYs, and *Z-ISO* varied from 26.58 in Valentine F1 to 263.97 in LA3004 with an average of 134.29 in HLYs (Figure S13). At the red ripe stage, the relative expression levels of *PSY1* ranged from 129.68 in Fla.8153 to 652.70 in AK-TC035 with an average of 378.81 in HLYs. *PDS* ranged from 2.70 in Fla.8153 to 14.04 in AK-TC035 with an average of 7.75 in HLYs, and *Z-ISO* varied from 61.71 in HLY13 to 311.47 in AK-TC035 with an average of 119.43 in HLYs (Figure S13).

### Relative expression levels of the downstream genes in HLYs at the breaker and red ripe stages

**Figure 6:**
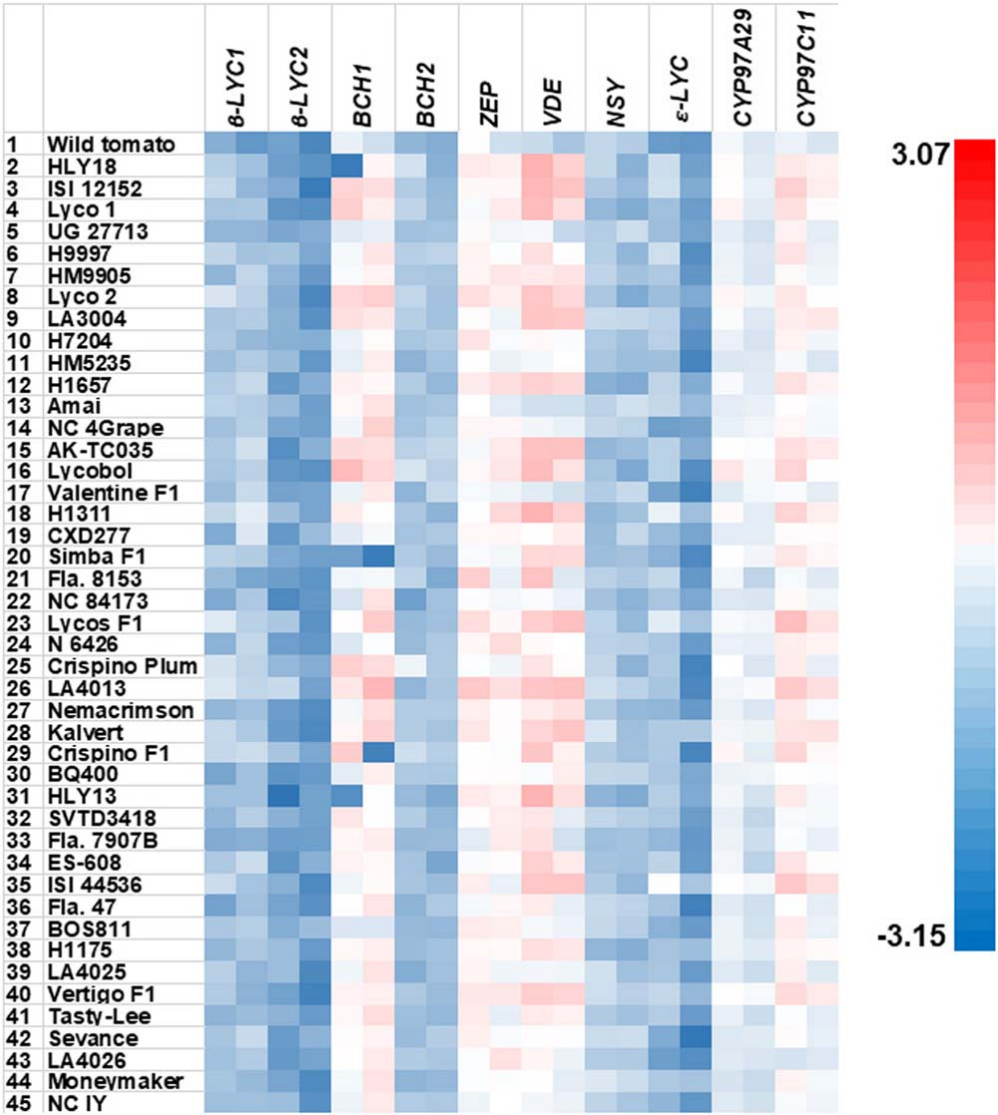
Heat map of relative expression levels of the ten downstream genes in the carotenoid biosynthesis pathway in the fruits of the 42 HLYs at the breaker and red ripe stages measured by qPCR. The relative expression of each gene was measured by qPCR, and relative quantification was performed using our newly published stepwise qPCR optimization method [49] with the tomato *Expressed* and *CAC* genes as the reference genes. The mean of the relative expression levels of the three biological replicates were log transformed. The wild tomato *S. pimpinellifolium* L. (LA2093) was used as the positive control genotype, Moneymaker was used a conventional low-lycopene control, and NC 1Y was used as the negative control. For each gene in each line, left and right boxes represent the breaker and red ripe stages, respectively.

Among the downstream genes in all genotypes, *BCH1*, *ZEP*, and *VDE* at both stages and *CYP97C11* at the breaker stage had relative expression levels larger than 1, indicating higher relative expression than the reference genes, while the other six downstream genes had relative expression levels smaller than 1, thus lower relative expression than the reference genes (Table 1; Figure 6). In HLYs, the average relative expression levels of *BCH1*, *ZEP*, *VDE* and *CYP97C11* (0.80–2.51) were comparable to the upstream genes *GGPPS2*, *PSY2* and *CrtISO-L1* (2.31–4.70), while which of *CYP97A29* (0.32–0.55) was comparable to the upstream genes *TPT1* and *CrtISO-L2* (0.39–0.64) (Table 1).

We compared the relative expression levels of all the downstream genes in HLYs as a group at breaker and red ripe stages with that of the controls at the same stages. We found that *β-LCY1*, *BCH1*, *BCH2*, *VDE*, *ε-LCY* and *CYP97C11* at both stages, and *β-LCY2* and *ZEP* at the red ripe stage had significantly higher relative expression levels in HLYs than their counterparts in the wild tomato (Table 1). Among these eight genes, we found that the relative expression levels of *β-LCY2* at both stages and *ε-LCY* at the red ripe stage in HLYs were insignificantly different from that of *crtiso* in NC 1Y (Table 1; Figures 6; S14). Therefore, silencing or minimal expression of two downstream genes (*β-LCY2* and *ε-LCY*) at either or both stages prevents further conversion of lycopene to carotenes. The significantly higher relative expression levels of seven downstream genes (*ε-LCY* at the breaker stage, *β-LCY1*, *BCH1*, *BCH2*, *VDE*, and *CYP97C11* at both stages, and *ZEP* at the red ripe stage) contributed to the lower fruit lycopene content in HLYs than in the wild tomato since higher expression of downstream genes might consume more lycopene. In addition, the relative expression levels of *β-LCY1*, *BCH2*, *NSY* and *ε-LCY* at both stages in HLYs as a group were extremely low (0.04–0.08; Table 1). In comparison to the wild tomato, however, the fold changes of the relative expression levels of *BCH1*, *VDE* and *CYP97C11* were 2.95, 20.92 and 3.90 in HLYs at the breaker stage, respectively, while that of *BCH1*, *ZEP* and *CYP97C11* were 9.44, 6.31 and 3.81 in HLYs at the red ripe stage, respectively. Thus, these four downstream genes (*BCH1*, *ZEP*, *VDE* and *CYP97C11*) largely contributed to the lower fruit lycopene content in HLYs than in the wild tomato. It is worthwhile to point out that the wild tomato had minimal relative expression levels in *β-LCY1*, *β-LCY2*, *BCH2* and *ε-LCY* at both stages and *VDE* and *NSY* at the red ripe stage, which ranged from 0.004 to 0.06 (Table 1).

When compared to Moneymaker, *BCH1* at the breaker stage, *BCH2*, *VDE*, *NSY* and *CYP97A29* at both stages, and *β-LCY1* at the red ripe stage had significantly higher relative expression levels in HLYs (Table 1). We also noticed that *BCH1* and *BCH2* had significantly increased relative expression levels at the red ripe stage than at the breaker stage in 8 and 3 out of the 42 HLYs, respectively. In contrast, *ε-LCY* and *CYP97A29* had significantly decreased relative expression levels at the red ripe stage than at the breaker stage in 7 and 5 out of the 42 HLYs, respectively (Figures 6; S14).

### Correlation coefficient analysis of fruit carotenoid content and the relative expression levels of the entire pathway genes at the red ripe stage

When comparing red ripe stage carotenoids with red ripe stage gene expression, correlation coefficient analysis showed that fruit lycopene content is positively correlated to upstream *ZDS* (*p* < 0.001), moderately positively with upstream *GGPPS2* (*p* < 0.1), but negatively correlated with upstream *GGPPS3*, *TPT2*, and downstream *ε-LCY* (*p* = 0.05 to 0.01; Table 2). β-carotene was positively correlated with downstream *ZEP*, *VDE*, and *CYP97C11* (*p* = 0.05 to 0.01), but negatively with upstream *TPT2* and downstream *CrtISO-L2* (*p* = 0.05 to 0.01) and moderately negatively with downstream *eLCY* (*p* < 0.1). Phytofluene was only positively correlated with upstream *PDS* (*p* = 0.01 to 0.001), negatively with *TPT2* and *PSY1* (*p* = 0.05 to 0.01), and moderately negatively with *GGPPS1* (*p* < 0.1; Table 2).

**Table 2:** Correlation coefficients of fruit carotenoid contents and gene relative expression levels at the red rip stage determined by simple linear regression correlation analysis

**Gene**	**Lycopene**	β**-carotene**	**Phytofluene**
Upstream genes			
*GGPPS1*	12.200	−4.690	−6.560^#^
*GGPPS2*	7.390^#^	0.0009	0.045
*GGPPS3*	−9.270^*^	−0.210	−0.170
*TPT1*	45.900	0.960	0.560
*TPT2*	−1312.000^*^	−78.700^*^	−36.600^*^
*SSU II*	1.350	−0.190	−0.036
*PSY1*	−0.080	−0.0002	−0.005^*^
*PSY2*	−2.410	0.150	0.042
*PSY3*	10.100	7.890	4.610
*PDS*	4.200	−0.280	0.290^**^
*Z-ISO*	−0.170	−0.004	0.0014
*ZDS*	5.020^***^	0.036	0.062
*CrtISO*	−0.390	0.045	−0.020
*CrtISO-L1*	−0.300	0.012	0.060
*CrtISO-L2*	−3.040	−2.480^*^	0.190
Downstream genes			
*β-LCY1*	−43.200	−0.170	−2.520
*β-LCY2*	73.600	45.800	−0.670
*BCH1*	−4.570	−0.210	−0.100
*BCH2*	−395.900	−11.500	5.300
*ZEP*	4.320	1.100^*^	0.390
*VDE*	4.320	1.320^*^	0.210
*NSY*	−50.400	7.510	5.520
ε-*LCY*	−0.060^*^	−3.160^#^	−11.700
*CYP97A29*	40.500	6.100^*^	−1.460
*CYP97C11*	−12.800	−1.400	−0.680

*^#^p*-value <0.1; ^*^p-value = 0.05 to 0.01; ^**^p-value = 0.01 to 0.001; and ^***^p-value <0.001.

## Discussion

**Figure 7:**
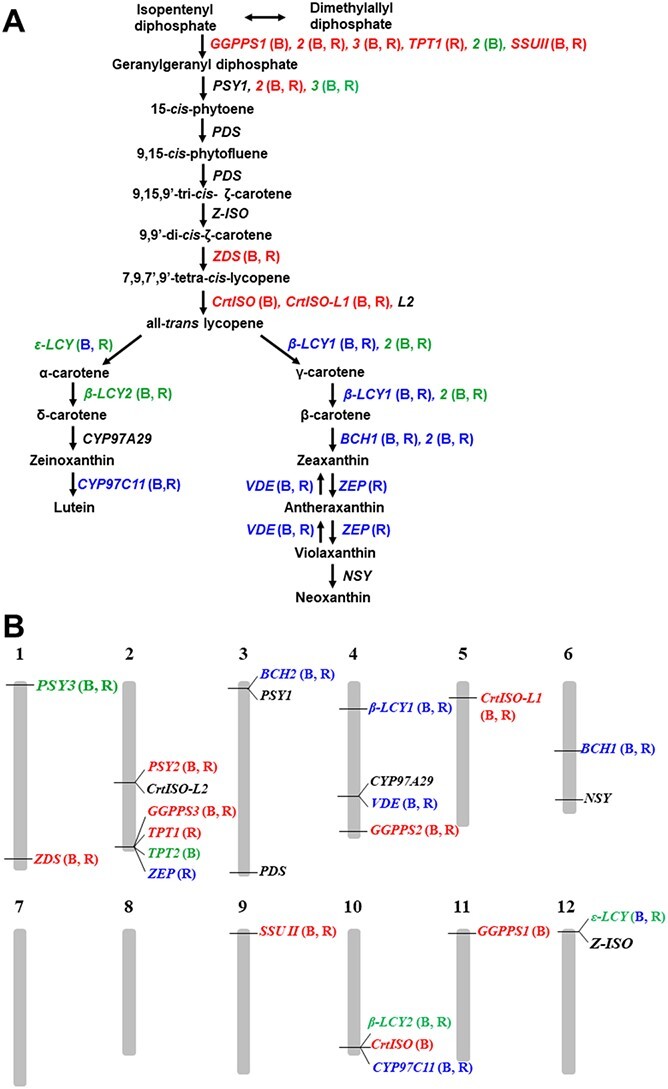
The carotenoid biosynthetic pathway in tomato (A) and chromosomal distribution of the 25 carotenoid biosynthesis pathway genes in tomato (B). The key carotenoid biosynthesis pathway genes (i.e. nine upstream genes (Red), seven downstream genes (blue) and two silenced or minimally expressed downstream genes (green)) affecting the fruit lycopene content in the red ripe fruits of the 42 potential HLYs were indicated. The 12 tomato chromosomes were labeled as 1 ~ 12 on the top of each chromosome. Red, significantly higher relative expression levels for nine upstream genes in comparison to Moneymaker; blue, significantly higher relative expression levels for seven downstream genes in comparison to the wild tomato *S. pimpinellifolium* L. (LA2093); green, insignificant difference in relative expression of four pathway genes in comparison to the non-functional *crtiso* in NC 1Y. Relative expression levels of each pathway gene were measured individually by qPCR with *Expressed* and *CAC* being the reference genes. Comparison was made to the low-lycopene control Moneymaker (for the upstream genes) or the positive control wild tomato (for the downstream genes). Statistics were conducted using two-tailed student’s *t*-test with two-sample unequal variance, i.e. significantly different expression from that in controls. B, breaker stage; R, red ripe stage.

Here we identified the key carotenoid biosynthesis pathway genes that contribute to high fruit lycopene content in the 42 potential HLYs. These key pathway genes include nine upstream genes (*GGPPS1*, *GGPPS2*, *GGPPS3*, *TPT1*, *SSU II*, *PSY2*, *ZDS*, *CrtISO* and *CrtISO-L1*), four downstream genes (*BCH1*, *ZEP*, *VDE* and *CYP97C11*) and two silenced or minimally expressed downstream genes (*β-LCY2* and *ε-LCY*) (Figure 7A; Table 1). These genes showed unique expression patterns prior to and after lycopene biosynthesis when compared to the controls. To the best of our knowledge, this is the first systematic investigation of the relative expression of the complete carotenoid biosynthesis pathway genes in various genotypes, and correlation of their expression patterns with fruit lycopene content.

The wild tomato had the highest amount of lycopene at the red ripe stage relative to all the other genotypes (Figures 3; 4). This is in accordance with Razifard et al. [32] who showed most of the selection pressure in tomato domestication was on fruit size rather than fruit color, leading to decreased fruit lycopene content in tomato breeding lines. Moneymaker had the lowest lycopene content besides NC 1Y (Figure 3).

Using qPCR, we found a general trend that most upstream genes were highly expressed and most downstream genes had very low expression in HLYs (plus the wild tomato) during fruit ripening, leading to high fruit lycopene content. This general trend has also been reported in the low-lycopene genotypes Moneymaker, M82, Tangerine 3183, and Rheilands Rhum as well as the high-lycopene genotypes Ailsa Craig and Red Setter [15, 17, 19, 20, 25, 59–63]. When compared to Moneymaker, we found higher expression in nine upstream genes at one or two stages (Table 1) contributed to the higher metabolic flux flow into the carotenoid biosynthesis pathway, leading to the high fruit lycopene content in HLYs. This is the first report of the relative expression levels of *TPT1*, *TPT2*, and *SSU II* in tomato during fruit ripening, while *GGPPS2* had been reported to have an increased expression in the orange fruits of Moneymaker [59].

During fruit ripening, it was reported that *PSY1* had increased expression in the low-lycopene genotypes M82 and Tangerine 3183 [17], Rheilands Rhum [61], and the high-lycopene genotypes Ailsa Craig [63] and Red Setter [20, 60]. Similarly, it was reported that *PDS* had enhanced expression in M82 and Tangerine 3183 [15, 17, 19], UC82-B [25] and Rheilands Rhum [61]. However, we found that *PSY1* at the red ripe stage and *PDS* at both stages did not significantly change expression in HLYs as a group in comparison to Moneymaker (Table 1; Figure S13). Expression of *PSY2* and *CrtISO* has been reported to be gradually increased during fruit ripening in M82 and Tangerine 3183 [17] and Red Setter [20, 60], but expression of *CrtISO-L1* and *ZDS* has never been reported during fruit ripening in tomato. In addition, we found the relative expression of downstream genes *CrtISO-L1* and *CrtISO-L2* at one or two stages was significantly higher than their counterparts in Moneymaker (Table 1; Figure S14).

Studies on expression of the downstream genes during fruit ripening were mainly conducted in low-lycopene genotypes such as M82 [15, 19], which differed in expression patterns from the present study. When compared to the wild tomato, we found a significant higher expression in seven downstream genes, i.e. *β-LCY1*, *BCH1*, *BCH2*, *ZEP*, *VDE*, *ε-LCY* and *CYP97C11* at one or two stages in HLYs (Table 1). Among these, higher expression of four downstream genes (*BCH1*, *ZEP*, *VDE* and *CYP97C11*) resulted in higher consumption of lycopene as a substrate for the downstream pathway, leading to lower fruit lycopene content in HLYs than in the wild tomato. Therefore, overexpression of upstream genes may not be necessarily increasing lycopene content as lycopene is exposed to increased pressure from downstream genes.

The higher expression in nine upstream genes enhanced metabolic flux from the upstream MEP pathway into the carotenoid biosynthetic pathway, while the silencing or minimal expression in two downstream genes prevents further conversion of lycopene to carotenes. Both of these mechanisms lead to enhanced fruit lycopene contents in HLYs than in Moneymaker. The higher expression of four key downstream genes also resulted in lower fruit lycopene content in HLYs than in the wild tomato. As shown in Figures 3 and 4, fruit lycopene and phytofluene contents showed a gradual increase across fruit ripening stages and reached the maximal levels at the red ripe stage in most genotypes, while fruit β-carotene contents showed a general decrease. The average fruit contents of lycopene and phytofluene in HLYs at both stages were significantly higher than that in Moneymaker, while the average fruit content of β-carotene in HLYs at both stages was comparable to that in Moneymaker, indicating that the high fruit lycopene content in HLYs did not occur at the expense of β-carotene content.

According to the correlation coefficient analysis of fruit carotenoid content and gene expression at the red ripe stage, the most significant gene was *ZDS*, which is positively correlated with increased lycopene content (Table 2). *ZDS* was also observed to be increased in expression in HLYs as compared to Moneymaker in both red ripe and breaker stages (Table 1; Fig. 7). *GGPPS3*, *TPT2*, and *ε-LCY* on the other hand were significantly negatively correlated with lycopene content even though *ε-LCY* is silenced (Table 2). *GGPPS2* showed a moderate (*p* = <0.1) positive relationship with lycopene content and was also upregulated in HLYs compared to Moneymaker at the same stage. Our correlation coefficient analysis did not identify all of the detected key pathway genes that affect fruit lycopene content, possibly because of the large variability in fruit carotenoid content from sample to sample for each HLY genotype.

The HLYs were developed using various genetic backgrounds, and the underlying mechanism for increased lycopene biosynthesis in most of them is largely unknown. Several genotypes are known for containing the *old-gold* (*og*) and *old gold*-*crimson* (*og^c^*) mutations in the *β–LCY2* promoter causing lowered *β–LCY2* expression, which leads to increased lycopene content at the expense of β-carotene [15, 64]. For example, LA4025 and LA4026 contained the *og* mutation while NC 4 Grape, Fla. 8153, SVTD3418, and Fla. 7907B harbored the *og^c^* mutation*.* We found that these genotypes were not significantly different in β-carotene from Moneymaker. We also found that *ε-LCY* at the red ripe stage and *β-LCY2* at both stages were minimally expressed or silenced in HLYs as a group when compared to the NC 1Y *crtiso* mutation (Table 1; Figures S13; S14). As a result, the *β-lcy2* mutants such as *old-gold* (*og*) and *old gold*-*crimson* (*og^c^*) and unknown *ε-lcy* mutant(s) had been widely used for the development of most, if not all, of the HLYs. According to Enfissi et al. [65], *og^c^* mutation resulted in higher gene expression in *GGPPS1, PDS*, *β-LCY1*, and *ε-LCY*, and lowered expression in *GGPPS2, PSY1, PSY2, ZDS, CrtISO*, and *β-LCY2* in low-lycopene genotypes, which were not observed in the present study (Figures 5; 6; S13; S14). In addition, a few genotypes such as LA3004 contained the *hp-1* mutation in the *DDB1* gene, and HLY18, HM5235, LA4013, and HLY13 harbored the *hp-2* mutation in *DET1*. Both *DDB1* and *DET1*, which do not belong to the carotenoid biosynthetic pathway, affect fruit lycopene content through the changes in chromoplast number and/or size [36, 66]. Kilambi et al. [67] found that an *hp-1* mutant had lowered expression in *GGPPS2*, *PSY1*, *PSY2*, *Z-ISO*, *CrtISO*, *β-LCY1,* and *β-LCY2*, and increased expression in *PDS*, *ZDS*, and *CYP97A29* at both maturity stages. Kolotilin et al. [36] reported that an *hp-2* mutant contained decreased expression of *β-LCY2* and enhanced expression in *GGPPS1* and *PDS* at the breaker stage as well as lowered expression of *BCH2* and increased expression of *GGPPS1*, *PSY1*, *PDS*, *ZDS* and *β-LCY2* at the red ripe stage. However, most of these were not observed in the *hp-1* mutant LA3004 and the *hp-2* mutants HLY18, HM5235, LA4013, and HLY13 (Figures S13; S14). These discrepancies in gene expression indicate that crossing of the original mutant genotypes with different breeding backgrounds dramatically changed expression levels of the pathway genes.

The chromosomal distribution of the nine upstream genes, seven downstream genes and the two silenced or minimal expressed genes (*β-LCY2* and *ε-LCY*) at either or both stages in HLYs clearly show the difficulty in improving tomato fruit lycopene content (Figure 7B). These genes should be the targets for genetic engineering, gene editing, and marker-assisted breeding to improve fruit lycopene content in tomato. Their promoters could also be engineered using gene editing [68, 69] or synthetic biology [70, 71] to fine-tune expression of these genes.

## Supplementary Material

Web_Material_uhac084

## Data Availability

All data were included in the paper and its Supplementary Materials published online.
