# Supplementary material for: Coordinated transcriptional regulation of the carotenoid biosynthesis contributes to fruit lycopene content in high-lycopene tomato genotypes
Source: Hortic Res. 2022 Jun 1;9:uhac084. doi: 10.1093/hr/uhac084 (PMC9160729; doi:10.1093/hr/uhac084)
Supplement: Web_Material_uhac084 [file web_material_uhac084.doc]

**Supplementary Material**

**Table S1.** **Tomato genotypes used in the present study.**

| **Genotype #** | **Name** | **Source^a^** | **Mutation(s)^b^** |
| --- | --- | --- | --- |
| 1 | Wild tomato (LA2093)**^c^** | TGRC (U.S.) | WT |
| 2 | HLY18 | Hazera Genetics (Israel) | *hp-2^dg^/hp-2^dg^* |
| 3 | ISI 12152 | Isi-Diamond (Italy) | N.A. |
| 4 | Lyco 1 | Hazera Genetics (Israel) | N.A. |
| 5 | UG 27713 | TGRC (U.S.) | N.A. |
| 6 | H9997 | Heinz Seeds (U.S.) | N.A. |
| 7 | HM9905 | H.M. Clause (France) | N.A. |
| 8 | Lyco 2 | Hazera Genetics (Israel) | N.A. |
| 9 | LA3004 | TGRC (U.S.) | *hp-1^w^/hp-1^w^* |
| 10 | H7204 | Heinz Seeds (U.S.) | N.A. |
| 11 | HM5235 | H.M. Clause (France) | *hp-2^dg^/hp-2^dg^* |
| 12 | H1657 | Heinz Seeds (U.S.) | N.A. |
| 13 | Amai | Sakata Seeds (Japan) | N.A. |
| 14 | NC 4Grape | NCTBP (U.S.) | *og^c^* |
| 15 | AK-TC035 | Akira Seeds (Spain) | N.A. |
| 16 | Lycobol | United Genetics (U.S.) | N.A. |
| 17 | Valentine F1 | Johnny Seeds (U.S.) | N.A. |
| 18 | H1311 | Heinz Seeds (U.S.) | N.A. |
| 19 | CXD277 | TGRC (U.S.) | N.A. |

**Table S1. (cont.)**

| **Genotype** **#** | **Name** | **Source^a^** | **Mutation(s)^b^** |
| --- | --- | --- | --- |
| 20 | Simba F1 | Isi-Diamond (Italy) | N.A. |
| 21 | Fla. 8153 | UFTBP (U.S.) | *og^c^* |
| 22 | NC 84173 | NCTBP (U.S.) | WT |
| 23 | Lycos F1 | Isi-Diamond (Italy) | N.A. |
| 24 | N 6426 | H.M. Clause (France) | N.A. |
| 25 | Crispino Plum | Esasem (Italy) | N.A. |
| 26 | LA4013 | TGRC (U.S.) | *hp-2* |
| 27 | Nemacrimson | United Genetics (U.S.) | N.A. |
| 28 | Kalvert | Esasem (Italy) | N.A. |
| 29 | Crispino F1 | Esasem (Italy) | N.A. |
| 30 | BQ400 | TGRC (U.S.) | N.A. |
| 31 | HLY13 | Hazera Genetics (Israel) | *hp-2^dg^/hp-2^dg^* |
| 32 | SVTD3418 | Seminis (U.S.) | *og^c^* |
| 33 | Fla. 7907B | UFTBP (U.S.) | *og^c^* |
| 34 | ES-608 | Esasem (Italy) | N.A. |
| 35 | ISI 44536 | Isi-Diamond (Italy) | N.A. |
| 36 | Fla. 47 | UFTBP (U.S) | N.A. |
| 37 | BOS811 | TGRC (U.S.) | N.A. |
| 38 | H1175 | Heinz Seeds (U.S.) | N.A. |
| 39 | LA4025 | TGRC (U.S.) | *og* |
| 40 | Vertigo F1 | Isi-Diamond (Italy) | N.A. |

**Table S1. (cont.)**

| **Genotype #** | **Name** | **Source^a^** | **Mutation(s)^b^** |
| --- | --- | --- | --- |
| 41 | Tasty-Lee Hybrid | Tasty-Lee (U.S.) | N.A. |
| 42 | Sevance | De Ruiter-Monsanto (U.S.) | N.A. |
| 43 | LA4026 | TGRC (U.S.) | *og* |
| 44 | Moneymaker | - | WT |
| 45 | NC IY | NCTBP (U.S.) | *tangerine* |

^a^TGRC, Tomato Genetics Resource Center; NCTBP, NC Tomato Breeding Program; UFTBP, UF Tomato Breeding Program. ^b^WT and w, wild-type; *og/og^c^*, *old-gold*/*old-gold crimson*; *hp-2^dg^*, *high-pigment-2* dark green. N.A., not available. ^c^Wild tomato (LA2093), *S. pimpinellifolium.*

**Table S2**. Primer sequences used in the present study.

| **Gene** | **Full Name** | **Accession #** | **Primer Name^a^** | **Primer Sequence**  **(5’ > 3’)** |
| --- | --- | --- | --- | --- |
| *GGPPS1* | *Gernanylgeranyl pyrophosphate 1* | Solyc11g011240.1.1 | F1 | GAAAGACAGCGGGTAAGGAC |
|  |  |  | **F2** | **AAAGACAGCGGGTAAGGACC** |
|  |  |  | R1 | CATTCATGGCCTTAGCCATC |
|  |  |  | **R2** | **AGCTCATTCATGGCCTTAGCC** |
| *GGPPS2* | *Gernanylgeranyl pyrophosphate 2* | Solyc04g079960.1.1 | **F1** | **GTTGATAAAACGACGTATCCG** |
|  |  |  | F2 | AAAACGACGTATCCGAAGCTGC |
|  |  |  | R1 | GTTGTTTAGCTTCGCCGTTG |
|  |  |  | **R2** | **AGCTGTTGTTTAGCTTCGC** |
| *GGPPS3* | *Gernanylgeranyl pyrophosphate 3* | Solyc02g085700.1.1 | F1 | GAAGGCATTTCTGATGTTGA |
|  |  |  | **F2** | **GAAGGCATTTCTGATGTTGAT** |
|  |  |  | **R1** | **CTCCTAATATAGCCCCTAGC** |
|  |  |  | R2 | CCTCCTAATATAGCCCCTAG |
| *TPT1* | *Trans-prenyltransferase 1* | Solyc02g085710.2.1 | F1 | AGCAATGATTGGTGGTGCG |
|  |  |  | **F2** | **AATGATTGGTGGTGCGTCC** |
|  |  |  | R1 | ACCTGAAACAGCAGTCCAAG |
|  |  |  | **R2** | **GTCATCCACAACCTGAAACAG** |
| *TPT2* | *Trans-prenyltransferase 2* | Solyc02g085720.1.1 | **F1** | **GTTGACATGCTGTGTGGAGA** |
|  |  |  | F2 | CATGCTGTGTGGAGATAAATG |
|  |  |  | **R1** | **CCAAGCAATGCTCCTACAAT** |
|  |  |  | R2 | TCAGATGCACCACCAAGCAA |

**Table S2. (Cont.)**

| **Gene** | **Full Name** | **Accession #** | **Primer Name** | **Primer Sequence**  **(5’ > 3’)** |
| --- | --- | --- | --- | --- |
| *SSU II* | *Small subunit II* | Solyc09g008920.2.1 | F1 | TGCTTCAGATGAGGAGATCC |
|  |  |  | **F2** | **CAGATGAGGAGATCCAACAC** |
|  |  |  | R1 | TCTCCGTCTTCTTTGCTTCC |
|  |  |  | **R2** | **TTCCCCTCAGTTTTGTTCTCC** |
| *PSY1* | *Phytoene synthase 1* | Solyc03g031860.2.1 | **F1:** | **TATTTGCTGGAAGGGTGACC** |
|  |  |  | F2: | CTGGAAGGGTGACCGATAAA |
|  |  |  | **R1:** | **CTGAGCTCAATTCTGTCACG** |
|  |  |  | R2: | TAGCTGAGCTCAATTCTGTC |
| *PSY2* | *Phytoene synthase 2* | Solyc02g081330.2.1 | F1 | TGTGAGCAAGCCAAAGAAGC |
|  |  |  | **F2** | **GAGCAAGCCAAAGAAGCTTC** |
|  |  |  | **R1** | **CTAGTGGGGAAGAAGTTGAC** |
|  |  |  | R2 | TGCTAGTGGGGAAGAAGTTG |
| *PSY3* | *Phytoene synthase 3* | Solyc01g005940.2.1 | **F1** | **ATGGAGAGAGTTCATGAAGG** |
|  |  |  | F2 | AGCAGATAAGAAGGGCAAGA |
|  |  |  | **R1** | **TGGCCAACGACTAGCTTTGT** |
|  |  |  | R2 | GATGACCATACTGGCCAACG |
| *PDS* | *Phytoene desaturase* | Solyc03g123760.2.1 | **F1** | **AGGAAAGCTTTGTGCTCAAG** |
|  |  |  | F2 | GGAAAGCTTTGTGCTCAAGC |
|  |  |  | **R1** | **AAACTACGCTTGCTTCCGAC** |
|  |  |  | R2 | CTAAACTACGCTTGCTTCCG |

**Table S2. (Cont.)**

| **Gene** | **Full Name** | **Accession #** | **Primer Name** | **Primer Sequence**  **(5’ > 3’)** |
| --- | --- | --- | --- | --- |
| *Z-ISO* | *ζ-carotene isomerase* | Solyc12g098710.1.1 | F1 | TGCAGCCATTCTTGATGGTC |
|  |  |  | **F2** | **AGCCATTCTTGATGGTCGTC** |
|  |  |  | **R1** | **GGAAGTAAGCACCTAATGTC** |
|  |  |  | R2 | GAGGAAGTAAGCACCTAATG |
| *ZDS* | *ζ-carotene desaturase* | Solyc01g097810.2.1 | **F1** | **TGTAATGTTGGAGAGCAGCTG** |
|  |  |  | F2 | AATGTTGGAGAGCAGCTGATG |
|  |  |  | **R1** | **CTCAACTCATCAGATAGGGAC** |
|  |  |  | R2 | GACTCAACTCATCAGATAGGG |
| *CrtISO* | *Carotenoid isomerase* | Solyc10g081650.1.1 | **F1** | **GTGTTGGCGATAGTTGCTTC** |
|  |  |  | F2 | GATAGTTGCTTCCCAGGAC |
|  |  |  | R1 | CCTAAGTCAGCTGCAACAC |
|  |  |  | **R2** | **TTCAAACCCTAAGTCAGCTG** |
| *CrtISO-L1* | *Carotenoid isomerase like 1* | Solyc05g010180.2.1 | F1 | CTTTTCCTGGCATTGGAGTTC |
|  |  |  | **F2** | **TTTCCTGGCATTGGAGTTCC** |
|  |  |  | R1 | AAGGCGCGAATGTTCTGAC |
|  |  |  | **R2** | **AGAAGGCGCGAATGTTCTG** |
| *CrtISO-L2* | *Carotenoid isomerase like 2* | Solyc02g085250.2.1 | F1 | ATCGGACTCCAATTGAAGGC |
|  |  |  | **F2** | **CCAATTGAAGGCCTATACTTG** |
|  |  |  | **R1** | **CTCAATGACAACATGCGCAG** |
|  |  |  | R2 | CTTGAAGTCCTCAATGACAAC |

**Table S2. (Cont.)**

| **Gene** | **Full Name** | **Accession #** | **Primer Name** | **Primer Sequence**  **(5’ > 3’)** |
| --- | --- | --- | --- | --- |
| *β-LCY1* | *Lycopene β-cyclase 1* | Solyc04g040190.1.1 | **F1** | **CTTCTGAAGCTTGATTTACCT** |
|  |  |  | F2 | TACCTGCTACAAGAAGGTTC |
|  |  |  | **R1** | **CAATCGAGACGATAAGAAG** |
|  |  |  | R2 | CAGGTAGAAACAATCGAGAC |
| *β-LCY2* | *Lycopene β-cyclase 2* | Solyc10g079480.1.1 | **F1** | **CTTCTGAAGCTTGATTTATCC** |
|  |  |  | F2 | CTGAAGCTTGATTTATCCGC |
|  |  |  | **R1** | **TGAGTTCAGGAAGAAACAGC** |
|  |  |  | R2 | CATGAGTTCAGGAAGAAACAG |
| *BCH1* | *β-carotene hydroxylase 1* | Solyc06g036260.2.1 | **F1** | **CCCATATGGCTTGTTCTTC** |
|  |  |  | F2 | GTTCTTCGGACCTAAGGAAC |
|  |  |  | **R1** | **CATGATCCTTTCGAAAGTCTC** |
|  |  |  | R2 | CGTTCATGATCCTTTCGAAAG |
| *BCH2* | *β-carotene hydroxylase 2* | Solyc03g007960.2.1 | **F1** | **CATAAGAGATTTCCCGTAGGG** |
|  |  |  | F2 | TAGGGCCTATTGCCAACGTG |
|  |  |  | **R1** | **GGGACACCATCAAATTTGTCC** |
|  |  |  | R2 | AAGCCATATGGGACACCATC |
| *ZEP* | *Zeaxanthin epoxidase* | Solyc02g090890.2.1 | F1 | CTTCTGAAAGGAAGGAAGAG |
|  |  |  | **F2** | **TCTGAAAGGAAGGAAGAGCG** |
|  |  |  | R1 | TGCTCAACGCCTGATGTTTG |
|  |  |  | **R2** | **AAATTGCTCAACGCCTGATG** |

**Table S2. (Cont.)**

| **Gene** | **Full Name** | **Accession #** | **Primer Name** | **Primer Sequence**  **(5’ > 3’)** |
| --- | --- | --- | --- | --- |
| *VDE* | *Violaxanthin de-epoxidase* | Solyc04g050930.2.1 | **F1** | **GAAAGTGGAAGAAGGAGAGC** |
|  |  |  | F2 | AAGAAGGAGAGCGGACAATC |
|  |  |  | R1 | AACCTTCGAACAGTCTACTG |
|  |  |  | **R2** | **GAAACCTTCGAACAGTCTAC** |
| *NSY* | *Neoxanthin synthase* | Solyc06g074240.1.1 | F1 | TCGGACATGGCTCAAACATG |
|  |  |  | **F2** | **CATGGCTCAAACATGACTAGG** |
|  |  |  | R1 | CTCTCTATTGCTAGATTGCC |
|  |  |  | **R2** | **GCTCTCTATTGCTAGATTGC** |
| *ε-LCY* | *Lycopene ε-cyclase* | Solyc12g008980.1.1 | F1 | CTTGGTTCAAGTCTTTCTTCAG |
|  |  |  | **F2** | **CAAGTCTTTCTTCAGCAGAC** |
|  |  |  | R1 | GCCTTTTCTCATGTCATTTGG |
|  |  |  | **R2** | **GATCAAGCCTTTTCTCATGTC** |
| *CYP97A29* | *Cytochrome P450-type monooxygenase 97A29* | Solyc04g051190.2.1 | **F1** | **TCAAATGGCTCTTGGAGCTC** |
|  |  |  | F2 | AAATGGCTCTTGGAGCTCC |
|  |  |  | **R1** | **TGGAGGTCTTGATCTTCGTG** |
|  |  |  | R2 | TGGGAACTATTGGAGGTCTTG |
| *CYP97C11* | *Cytochrome P450-type monooxygenase 97C11* | Solyc10g083790.1.1 | **F1** | **GTTGGAAGCTACAATTGCTC** |
|  |  |  | F2 | TGGAAGCTACAATTGCTCTC |
|  |  |  | R1 | GTTGCTCCAGTAGTCATGC |
|  |  |  | **R2** | **TGGTTGCTCCAGTAGTCATG** |

**Table S2. (Cont.)**

| **Gene** | **Full Name** | **Accession #** | **Primer Name** | **Primer Sequence**  **(5’ > 3’)** |
| --- | --- | --- | --- | --- |
| *CAC* (Control) | *Clathrin adaptor complexes medium subunit* | Solyc08g006960.2.1 | F1 | AAAAGTCCTTGACTCGTCCG |
|  |  |  | **F2** | **TGACTCGTCCGCCAATTCAA** |
|  |  |  | **R1** | **TCTCCCACACCTTGAGAAAC** |
|  |  |  | R2 | TTGTAGCCACTCTTCTCCCAC |
| *Expressed* (Control) | *Expressed sequence* | Solyc07g025390.2.1 | **F1** | **CACACCCAAATGCACCAGTT** |
|  |  |  | F2 | ACACCCAAATGCACCAGTTG |
|  |  |  | R1 | CACCGTAACACAATGGAAGC |
|  |  |  | **R2** | **ACACCGTAACACAATGGAAG** |

^a^The best primer pair for each gene was in bold and used for qPCR for the measurement of the relative expression levels of each gene.

**Table S3. Optimized qPCR conditions for the 25 carotenoid biosynthetic pathway genes and the 2 reference genes (*Expressed* and *CAC*) in the red ripe fruits of the genotype Amai.**

| **Gene** | **Primer Pair** | **Amplicon**  **Length (bp)** | **Optimal Tm (⁰C)** | **Optimal Primer Conc. (nM)** | **cDNA**  **Dilution** | **R^2^** | **E (%)** |
| --- | --- | --- | --- | --- | --- | --- | --- |
| *GGPPS1* | F1/R1^a^ | - | - | - | - | - | - |
|  | F1/R2 | 115 | 59.0 | 350 | 1 – 1/16 | 0.9888 | 101.8 |
|  | F2/R1 | - | - | - | - | - | - |
|  | **F2/R2**^b^ | **114** | **59.0** | **300** | **1/5 – 1/20** | **1.000** | **99.5** |
| *GGPPS2* | F1/R1 | 85 | 59.0 | 350 | 1/10 – 1/160 | 0.9996 | 93.6 |
|  | **F1/R2** | **89** | **59.0** | **350** | **1/10 – 1/160** | **0.9960** | **99.1** |
|  | F2/R1 | 79 | 59.0 | 300 | 1/10 – 1/160 | 0.9963 | 88.8 |
|  | F2/R2 | 83 | 59.0 | 300 | 1/10 – 1/160 | 0.9986 | 95.0 |
| *GGPPS3* | F1/R1 | 103 | 56.8 | 350 | 1/10 – 1/160 | 0.9908 | 112.8 |
|  | F1/R2 | 104 | 56.8 | 350 | 1/10 – 1/160 | 0.9998 | 89.0 |
|  | **F2/R1** | **103** | **59.0** | **350** | **1/10 – 1/80** | **0.9998** | **97.4** |
|  | F2/R2 | 104 | 56.8 | 350 | 1/10 – 1/160 | 0.9964 | 94.5 |
| *TPT1* | F1/R1 | 81 | 56.8 | 350 | 1/40 – 1/160 | 1.000 | 109.8 |
|  | F1/R2 | 91 | 56.8 | 300 | 1/10 – 1/80 | 0.9979 | 93.9 |
|  | F2/R1 | 78 | 56.8 | 350 | 1/20 – 1/160 | 0.9997 | 110.7 |
|  | **F2/R2** | **88** | **59.0** | **300** | **1/10 – 1/80** | **0.9986** | **97.0** |
| *TPT2* | **F1/R1** | **116** | **59.0** | **350** | **1/5 – 1/80** | **0.9912** | **103.4** |
|  | F1/R2 | 128 | 59.0 | 300 | 1 – 1/16 | 0.9919 | 94.3 |

**Table S3. (Cont.)**

| **Gene** | **Primer Pair** | **Amplicon**  **Length (bp)** | **Optimal Tm (⁰C)** | **Optimal Primer Conc. (nM)** | **cDNA**  **Dilution** | **R^2^** | **E (%)** |
| --- | --- | --- | --- | --- | --- | --- | --- |
|  | F2/R1 | 111 | 59.0 | 350 | 1/10 – 1/160 | 0.9593 | 80.6 |
|  | F2/R2 | 121 | 59.0 | 300 | 1/10 – 1/160 | 0.9953 | 98.6 |
| *SSU II* | F1/R1 | 101 | 56.8 | 350 | 1/10 – 1/160 | 0.9654 | 139.7 |
|  | F1/R2 | 117 | 56.8 | 300 | 1/10 – 1/160 | 0.9920 | 108.8 |
|  | F2/R1 | 96 | 56.8 | 350 | 1/10 – 1/160 | 0.9849 | 111.8 |
|  | **F2/R2** | **112** | **59.0** | **350** | **1/10 – 1/40** | **0.9999** | **101.5** |
| *PSY1* | **F1/R1** | **111** | **59.0** | **300** | **1/20 – 1/80** | **0.9988** | **99.3** |
|  | F1/R2 | 114 | 59.0 | 300 | 1/20 – 1/160 | 0.9942 | 86.3 |
|  | F2/R1 | 105 | 59.0 | 300 | 1/10 – 1/80 | 0.9998 | 92.4 |
|  | F2/R2 | 108 | 59.0 | 300 | 1/10 – 1/80 | 0.9941 | 96.85 |
| *PSY2* | F1/R1 | 86 | 56.8 | 350 | 1/10 – 1/160 | 0.9967 | 103.3 |
|  | F1/R2 | 88 | 56.8 | 300 | 1/10 – 1/160 | 0.9997 | 85.2 |
|  | **F2/R1** | **83** | **59.0** | **350** | **1/10 – 1/160** | **0.9988** | **102.3** |
|  | F2/R2 | 85 | 56.8 | 300 | 1/10 – 1/160 | 0.9978 | 88.9 |
| *PSY3* | **F1/R1** | **100** | **59.0** | **300** | **1/10 – 1/80** | **0.9941** | **98.5** |
|  | F1/R2 | - | - | - | - | - | - |
|  | F2/R1 | 80 | 56.8 | 350 | 1/20 – 1/160 | 0.9833 | 90.1 |
|  | F2/R2 | - | - | N/A | - | - | - |
| *PDS* | **F1/R1** | **89** | **59.0** | **350** | **1/10 – 1/160** | **0.9955** | **99.2** |

**Table S3. (Cont.)**

| **Gene** | **Primer Pair** | **Amplicon**  **Length (bp)** | **Optimal Tm (⁰C)** | **Optimal Primer Conc. (nM)** | **cDNA**  **Dilution** | **R^2^** | **E (%)** |
| --- | --- | --- | --- | --- | --- | --- | --- |
|  | F1/R2 | 91 | 59.0 | 350 | 1/10 – 1/160 | 0.9998 | 87.0 |
|  | F2/R1 | 88 | 59.0 | 300 | 1/10 – 1/160 | 0.9818 | 90.0 |
|  | F2/R2 | 90 | 59.0 | 350 | 1/10 – 1/160 | 0.9998 | 89.1 |
| *Z-ISO* | F1/R1 | 107 | 56.8 | 350 | 1/10 - 1/160 | 0.9987 | 105.2 |
|  | F1/R2 | 109 | 56.8 | 350 | 1/10 – 1/160 | 0.9974 | 98.2 |
|  | **F2/R1** | **104** | **59.0** | **350** | **1/5 – 1/80** | **0.9983** | **99.5** |
|  | F2/R2 | 106 | 56.8 | 300 | 1/100 – 1/1600 | 0.9928 | 91.1 |
| *ZDS* | **F1/R1** | **98** | **59.0** | **300** | **1/10 – 1/160** | **0.9988** | **99.1** |
|  | F1/R2 | 100 | 56.8 | 300 | 1/10 – 1/160 | 0.9994 | 93.3 |
|  | F2/R1 | 95 | 56.8 | 300 | 1/10 – 1/160 | 0.9995 | 104.4 |
|  | F2/R2 | 97 | 56.8 | 300 | 1/10 – 1/160 | 0.9994 | 94.8 |
| *CrtISO* | F1/R1 | 91 | 59.0 | 350 | 1/10 – 1/80 | 0.9992 | 111.6 |
|  | **F1/R2** | **98** | **59.0** | **350** | **1/10 – 1/40** | **0.9997** | **105.0** |
|  | F2/R1 | 83 | 59.0 | 350 | 1/10 – 1/80 | 0.9971 | 86.7 |
|  | F2/R2 | 90 | 59.0 | 300 | 1/10 – 1/80 | 0.9993 | 110.5 |
| *CrtISO-L1* | F1/R1 | 89 | 59.0 | 300 | 1/10 – 1/160 | 0.9954 | 99.3 |
|  | F1/R2 | 91 | 59.0 | 300 | 1/10 – 1/160 | 0.9939 | 91.9 |
|  | F2/R1 | 87 | 59.0 | 350 | 1/10 – 1/160 | 0.9972 | 96.8 |
|  | **F2/R2** | **89** | **59.0** | **350** | **1/10 – 1/160** | **0.9985** | **100.9** |

**Table S3. (Cont.)**

| **Gene** | **Primer Pair** | **Amplicon**  **Length (bp)** | **Optimal Tm (⁰C)** | **Optimal Primer Conc. (nM)** | **cDNA**  **Dilution** | **R^2^** | **E (%)** |
| --- | --- | --- | --- | --- | --- | --- | --- |
| *CrtISO-L2* | F1/R1 | 104 | 59.0 | 300 | 1/10 – 1/160 | 0.9902 | 111.1 |
|  | F1/R2 | 113 | 59.0 | 350 | 1/10 – 1/160 | 0.9983 | 90.7 |
|  | **F2/R1** | **96** | **59.0** | **350** | **1/10 – 1/160** | **0.9983** | **100.6** |
|  | F2/R2 | 105 | 59.0 | 350 | 1/10 – 1/160 | 0.9992 | 95.1 |
| *β-LCY1* | **F1/R1** | **85** | **59.0** | **350** | **1/10 – 1/160** | **0.9987** | **99.0** |
|  | F1/R2 | 88 | 56.8 | 350 | 1/10 – 1/160 | 0.9984 | 93.9 |
|  | F2/R1 | 77 | 56.8 | 350 | 1/10 – 1/160 | 0.9902 | 92.3 |
|  | F2/R2 | 80 | 56.8 | 350 | 1/10 – 1/160 | 0.9989 | 96.0 |
| *β-LCY2* | **F1/R1** | **111** | **59.0** | **350** | **1/5 – 1/80** | **0.9954** | **100.1** |
|  | F1/R2 | 121 | 56.8 | 350 | 1/5 – 1/80 | 0.9963 | 90.5 |
|  | F2/R1 | 109 | 56.8 | 350 | 1/5 – 1/80 | 0.9950 | 99.7 |
|  | F2/R2 | 119 | 56.8 | 350 | 1/5 – 1/80 | 0.9988 | 93.6 |
| *BCH1* | **F1/R1** | **105** | **59.0** | **300** | **1/20 – 1/160** | **0.9993** | **98.0** |
|  | F1/R2 | 109 | 59.0 | 350 | 1/10 – 1/160 | 0.9977 | 105.5 |
|  | F2/R1 | 93 | 59.0 | 350 | 1/10 – 1/160 | 0.9859 | 87.7 |
|  | F2/R2 | 97 | 59.0 | 350 | 1/10 – 1/160 | 0.9805 | 104.0 |
| *BCH2* | **F1/R1** | **101** | **59.0** | **300** | **1/20 – 1/80** | **0.9977** | **97.3** |
|  | F1/R2 | 110 | 59.0 | 300 | 1/10 – 1/40 | 0.9971 | 96.2 |
|  | F2/R1 | - | - | - | - | - | - |

**Table S3. (Cont.)**

| **Gene** | **Primer Pair** | **Amplicon**  **Length (bp)** | **Optimal Tm (⁰C)** | **Optimal Primer Conc. (nM)** | **cDNA**  **Dilution** | **R^2^** | **E (%)** |
| --- | --- | --- | --- | --- | --- | --- | --- |
|  | F2/R2 | - | - | - | - | - | - |
| *ZEP* | F1/R1 | - | - | - | - | - | - |
|  | F1/R2 | - | - | - | - | - | - |
|  | F2/R1 | 105 | 59.0 | 350 | 1/10 – 1/160 | 0.9945 | 89.5 |
|  | **F2/R2** | **109** | **59.0** | **350** | **1/10 – 1/40** | **0.9991** | **100.0** |
| *VDE* | F1/R1 | 113 | 56.8 | 350 | 1/10 – 1/160 | 0.9926 | 117.2 |
|  | F1/R2 | 115 | 59.0 | 350 | 1/5 – 1/80 | 0.9969 | 95.5 |
|  | **F2/R1** | **105** | **59.0** | **350** | **1/10 – 1/80** | **0.9952** | **97.0** |
|  | F2/R2 | 107 | 56.8 | 350 | 1/10 – 1/160 | 0.9936 | 95.1 |
| *NSY* | F1/R1 | 91 | 56.8 | 300 | 1/5 – 1/80 | 0.9537 | 101.76 |
|  | F1/R2 | 92 | 56.8 | 300 | 1/5 - 1/80 | 0.9894 | 87.5 |
|  | F2/R1 | 86 | 56.8 | 300 | 1/5 – 1/80 | 0.9973 | 111.5 |
|  | **F2/R2** | **87** | **59.0** | **300** | **1/5 – 1/80** | **0.9976** | **100.8** |
| *ε-LCY* | F1/R1 | 84 | 56.8 | 300 | 1/20 – 1/320 | 0.9537 | 101.8 |
|  | F1/R2 | 90 | 56.8 | 300 | 1/20 – 1/320 | 0.9894 | 87.5 |
|  | F2/R1 | 77 | 56.8 | 300 | 1/20 – 1/320 | 0.9973 | 111.5 |
|  | **F2/R2** | **83** | **59.0** | **300** | **1/20 – 1/80** | **0.9995** | **100.8** |
| *CYP97A29* | **F1/R1** | **103** | **59.0** | **350** | **1/10 – 1/160** | **0.9986** | **102.7** |
|  | F1/R2 | 113 | 59.0 | 350 | 1/10 – 1/160 | 0.9964 | 89.2 |

**Table S3. (Cont.)**

| **Gene** | **Primer Pair** | **Amplicon**  **Length (bp)** | **Optimal Tm (⁰C)** | **Optimal Primer Conc. (nM)** | **cDNA**  **Dilution** | **R^2^** | **E (%)** |
| --- | --- | --- | --- | --- | --- | --- | --- |
|  | F2/R1 | 101 | 59.0 | 350 | 1/10 – 1/160 | 0.9996 | 107.9 |
|  | F2/R2 | 111 | 59.0 | 350 | 1/10 – 1/160 | 0.9897 | 111.1 |
| *CYP97C11* | F1/R1 | 93 | 56.8 | 350 | 1/10 – 1/160 | 0.9863 | 130.2 |
|  | **F1/R2** | **95** | **59.0** | **300** | **1/10 – 1/160** | **0.9974** | **103.6** |
|  | F2/R1 | 91 | 56.8 | 350 | 1/10 – 1/160 | 0.9996 | 115.5 |
|  | F2/R2 | 93 | 56.8 | 300 | 1/10 – 1/160 | 0.9974 | 96.0 |
| *CAC* (Control) | F1/R1 | 96 | 56.8, 59.0 | 350 | 1/10 - 1/160 | 0.9994 | 110.5 |
|  | F1/R2 | 109 | 56.8, 59.0 | 350 | 1/10 - 1/160 | 0.9989 | 95.2 |
|  | **F2/R1** | **87** | **59.0** | **350** | **1/10 – 1/160** | **0.9989** | **98.6** |
|  | F2/R2 | 100 | 56.8, 59.0 | 350 | 1/10 - 1/160 | 0.9969 | 97.96 |
| *Expressed* (Control) | F1/R1 | 110 | 56.8, 59.0 | 350 | 1/10 – 1/160 | 0.9985 | 105.6 |
|  | **F1/R2** | **111** | **59.0** | **250** | **1/10 – 1/160** | **0.9951** | **100.2** |
|  | F2/R1 | 109 | 56.8, 59.0 | 350 | 1/10 – 1/160 | 0.9919 | 111.5 |
|  | F2/R2 | 110 | 56.8, 59.0 | 250 | 1/10 – 1/160 | 0.9983 | 111.2 |
|  | F1/R2 | 113 | 59.0 | 350 | 1/10 – 1/160 | 0.9964 | 89.2 |

^a^The primer pair failed for qPCR. ^b^The best primer pair for each gene was in bold and used for qPCR for the measurement of the relative expression levels of each gene.

**Figure S1. Fruit contents of lycopene, *β*-carotene and phytofluene in the wild tomato *S. pimpinellifolium* L. (LA2093), the average of the 42 potential HLYs, Moneymaker and NC 1Y at the pink (A) and red ripe (B) stages.** All genotypes were grown together under the same greenhouse conditions. Contents of trans-lycopene, *β*-carotene and phytofluene content of pericarp tissue on a fresh weight (FW) was determined using HPLC. *denotes *p*-value < 0.05 using a two-tailed student’s t-test with two-sample unequal variance.

**
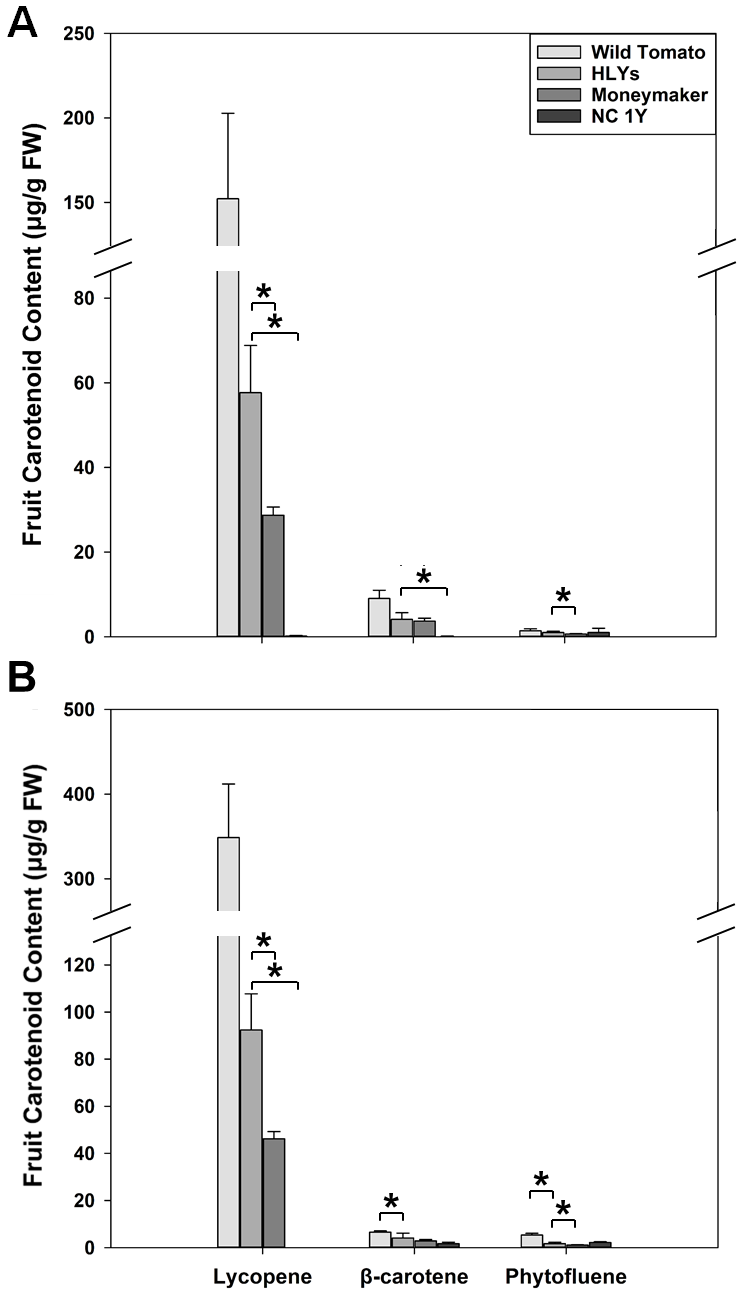
**

**Figure S2. Phylogenetic tree of the carotenoid biosynthesis genes and the reference genes in tomato.** The tree was constructed using the maximum likelihood method and the full-length protein sequences of those genes and tested with 1,000 bootstrap replications. Bootstrap values are presented at their corresponding nodes. The cDNA sequence alignment of the grouped genes on the right were used for sequence-specific primer design.


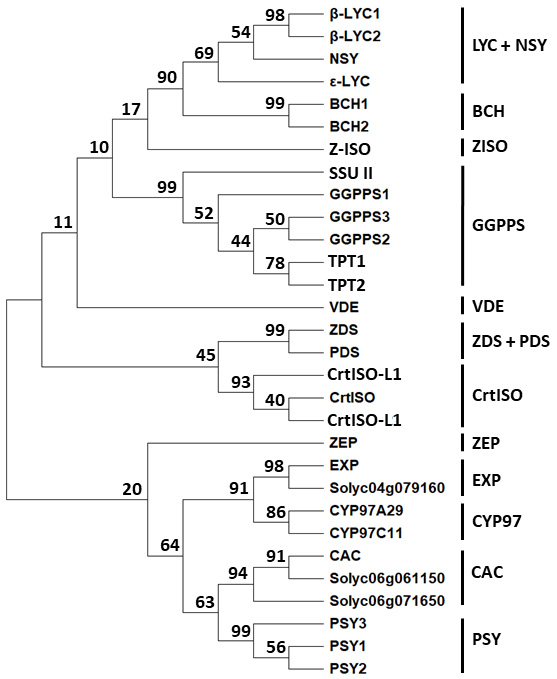


**Figure S3.** **Alignment of the** **cDNA sequences of the tomato *GGPPS1-3, TPT1, TPT2* and *SSUII* genes for qPCR primer design.**


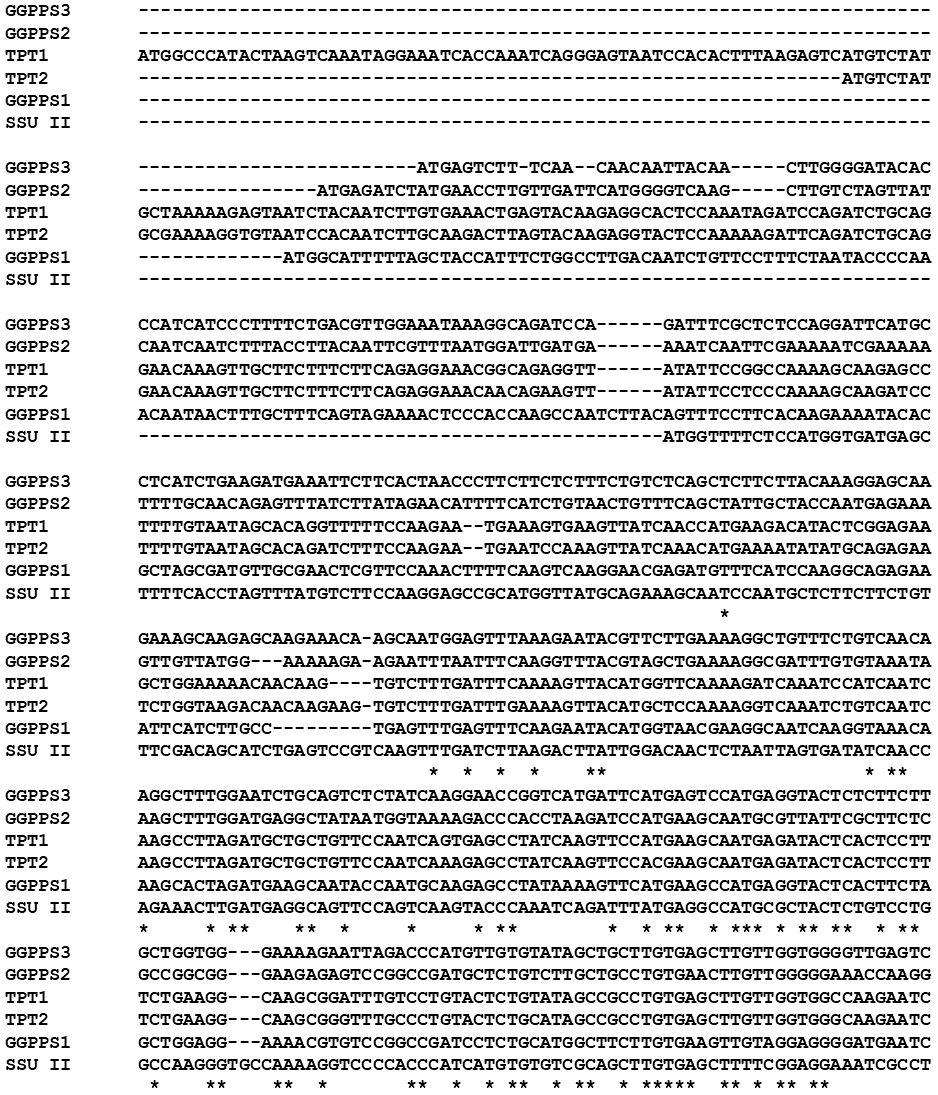


**Figure S3. (Cont.)**

**
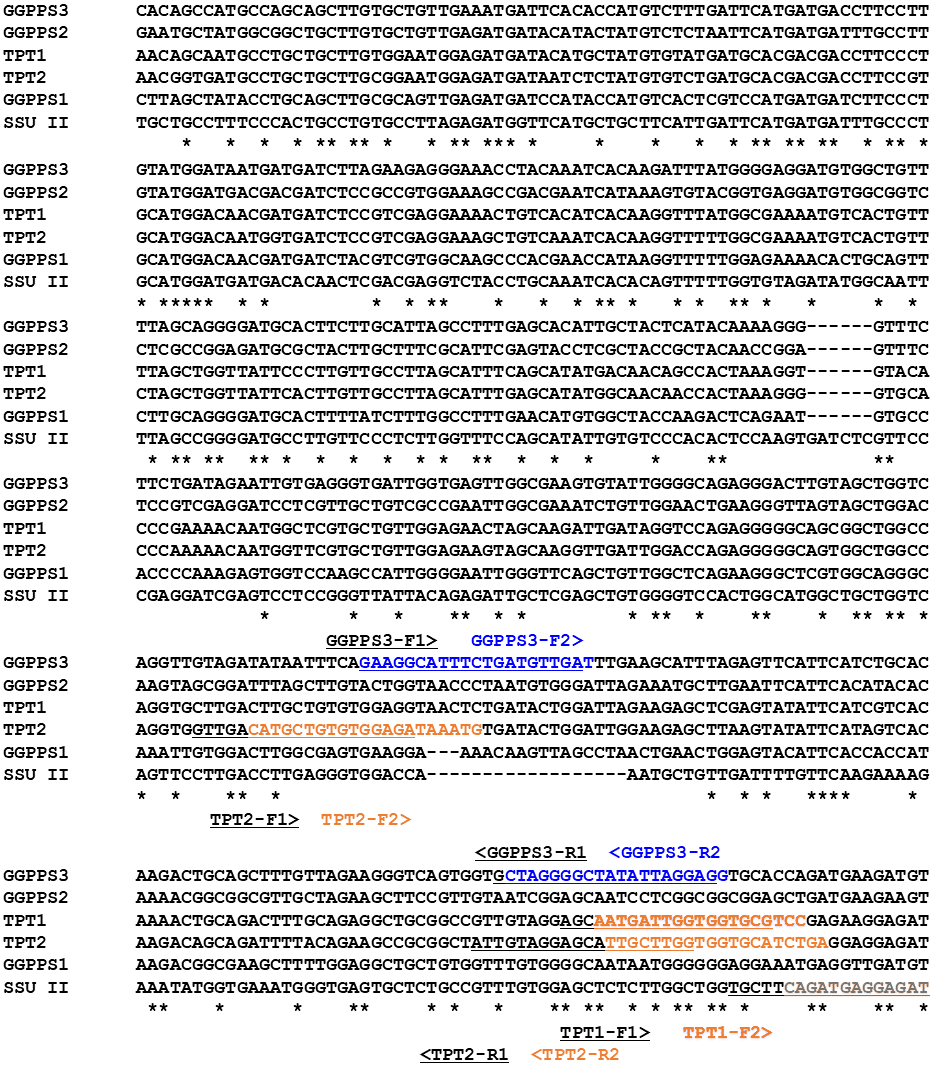
**

**Figure S3. (Cont.)**


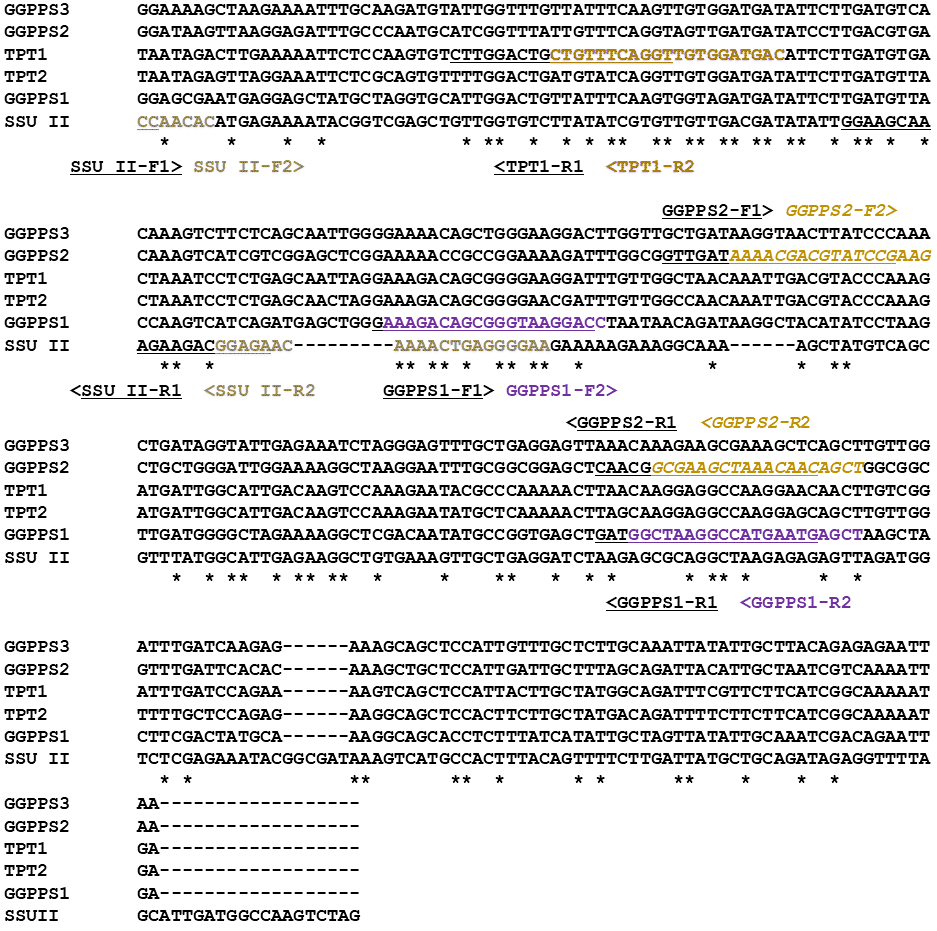


**Figure S4.** **Alignment of the** **cDNA sequences of the tomato *PSY1-3* genes for qPCR primer design.**


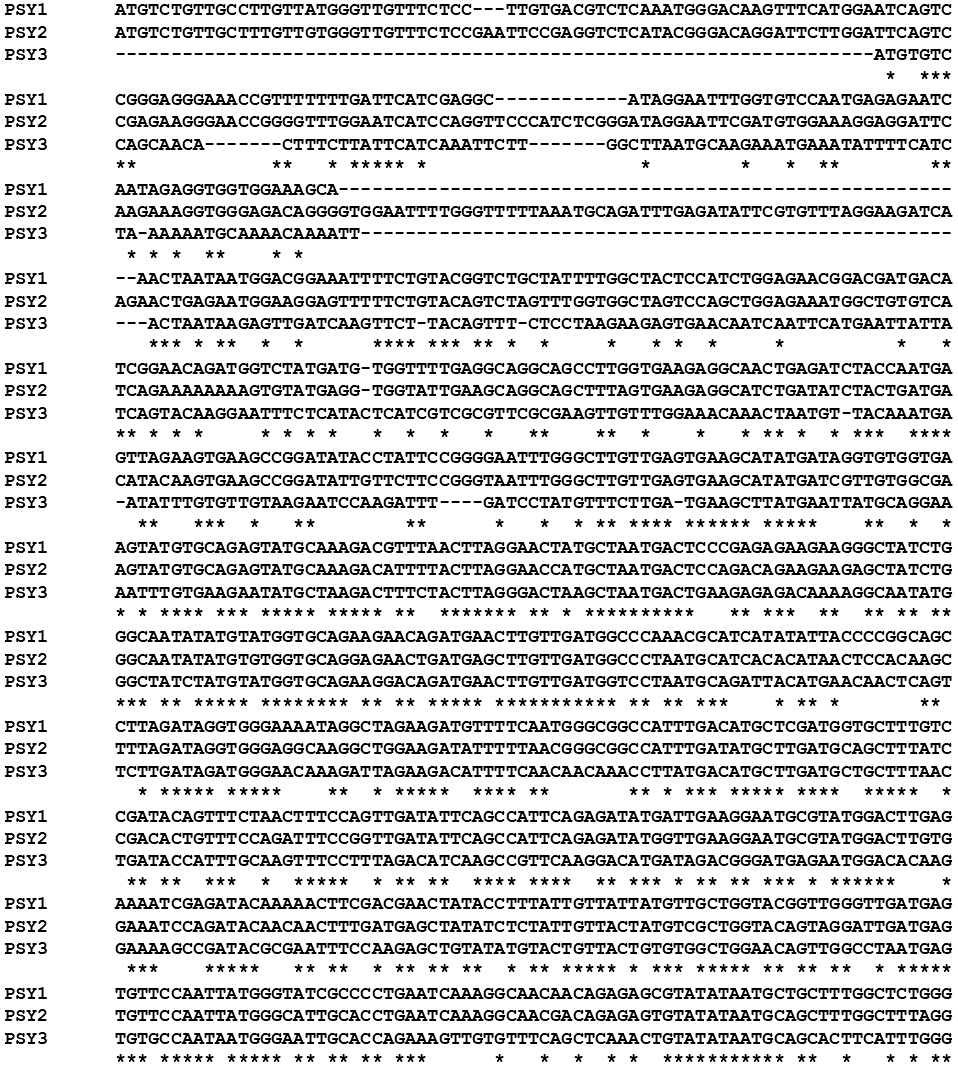


**Figure S4. (Cont.)**

**
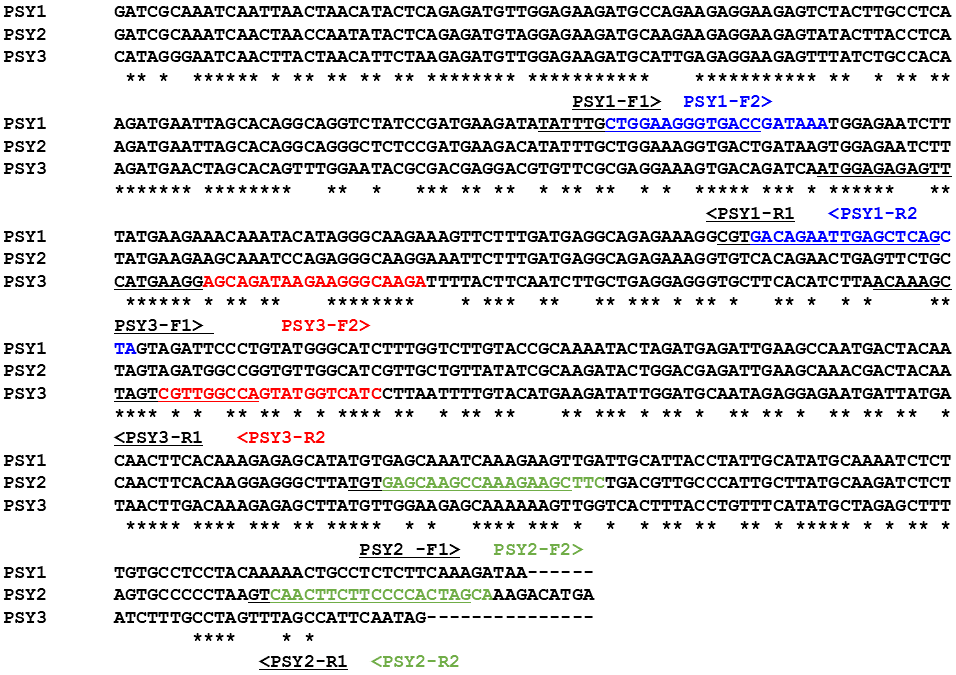
**

**Figure S5.** **Alignment of the** **cDNA sequences of the tomato *PDS* and *ZDS* genes for qPCR primer design.**

**
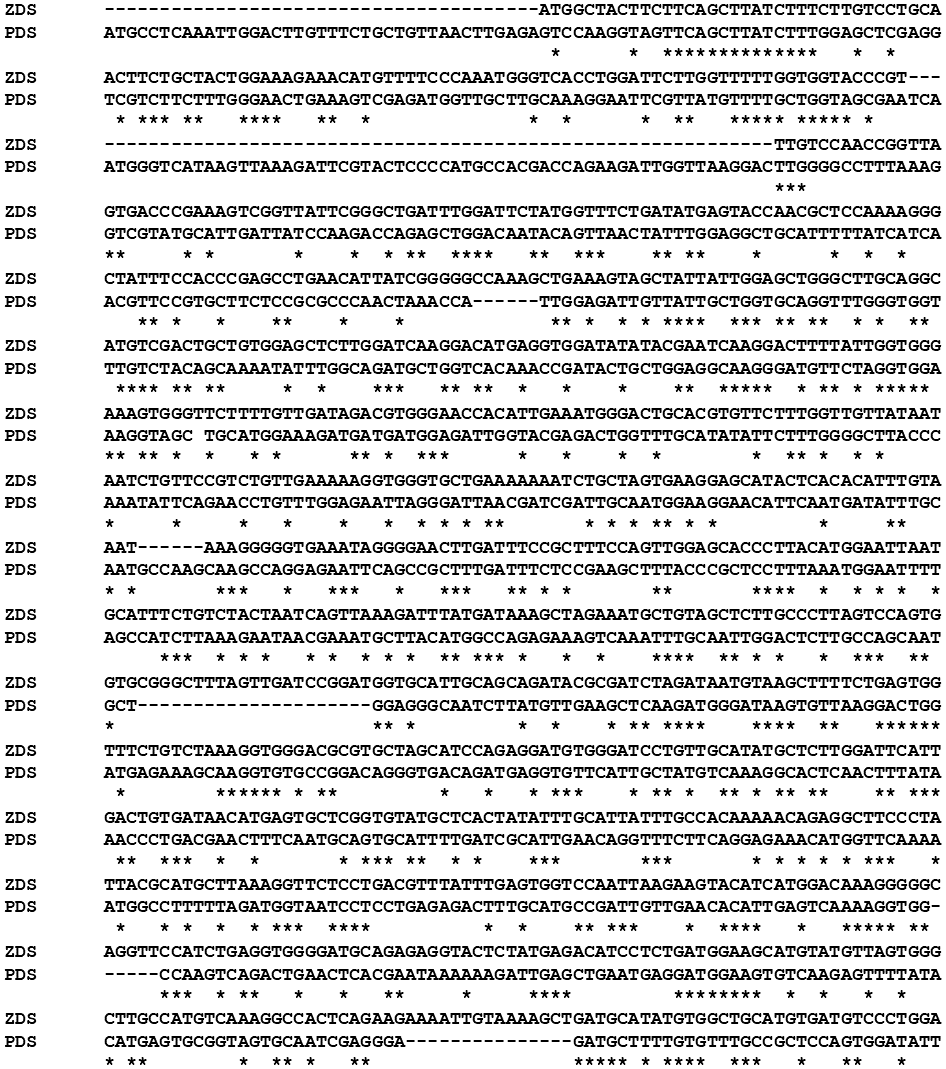
**

**Figure S5. (Cont.)**


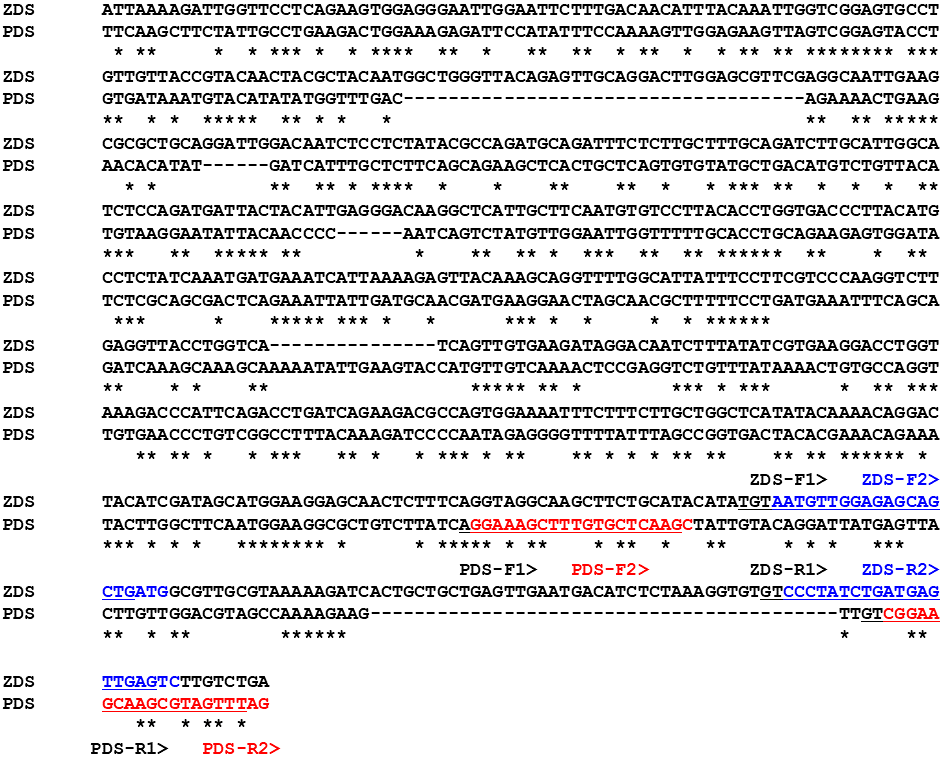


**Figure S6.** **The** **cDNA sequence of the tomato *Z-ISO* gene for qPCR primer design.**


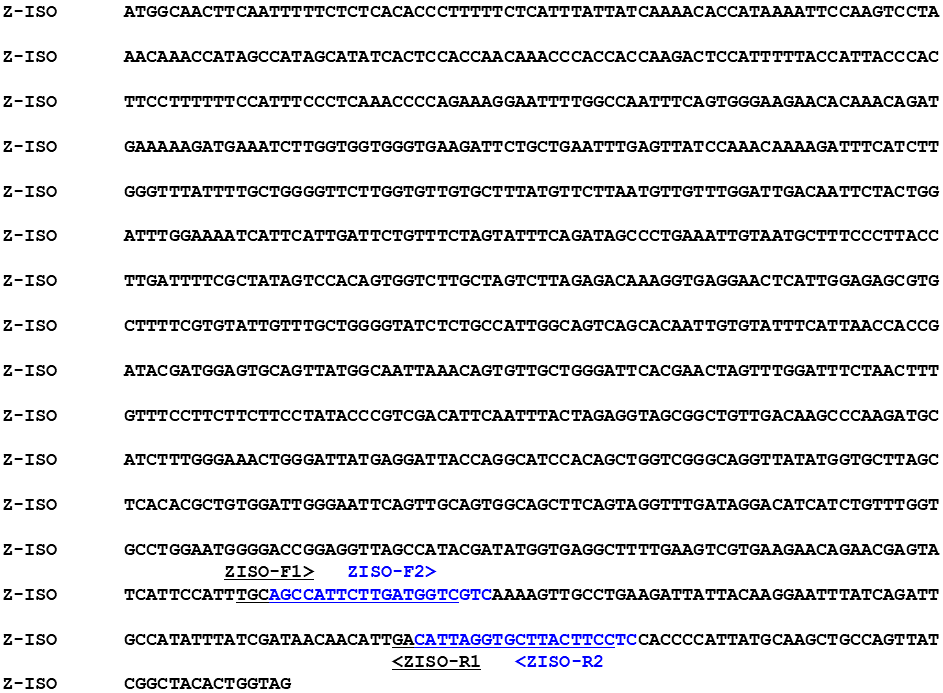


**Figure S7.** **Alignment of the** **cDNA sequences of the tomato *CrtISO*, *CrtISO-L1* and *CrtISO-L2* genes for qPCR primer design.**


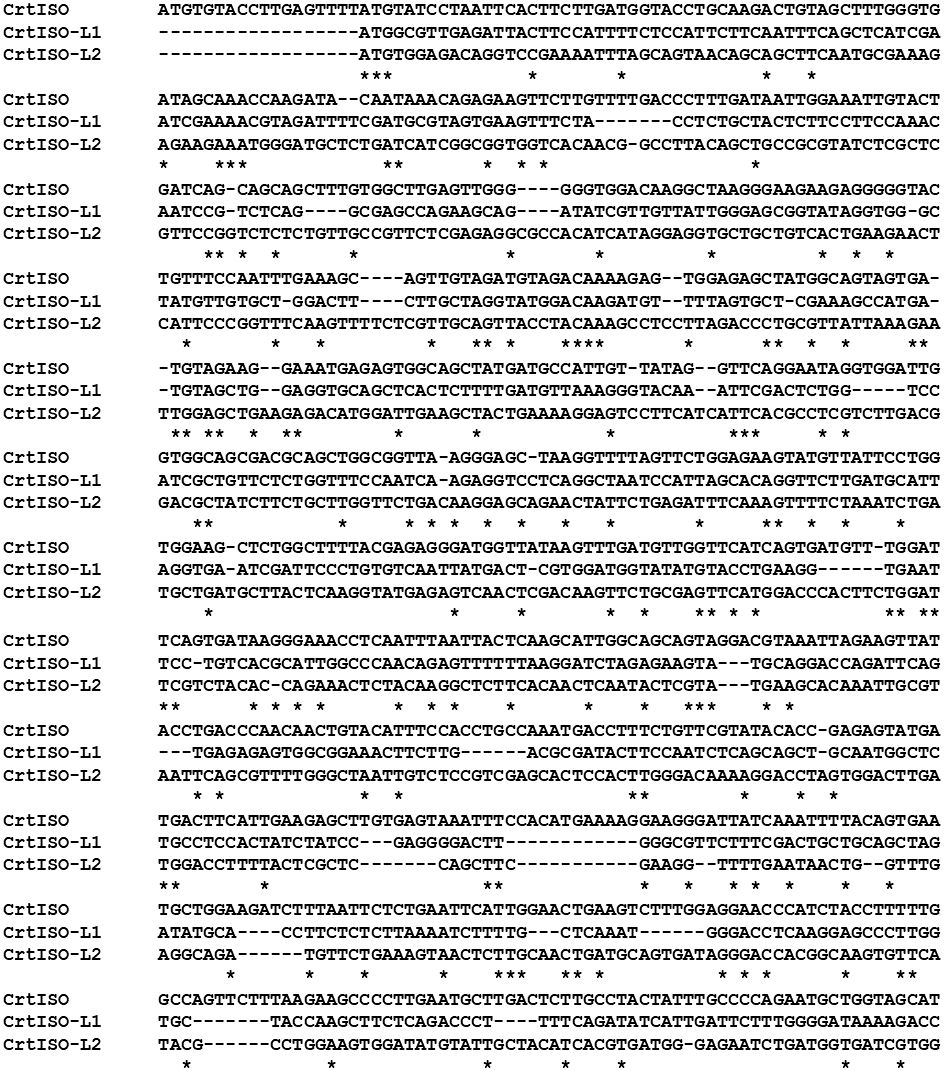


**Figure S7. (Cont.)**


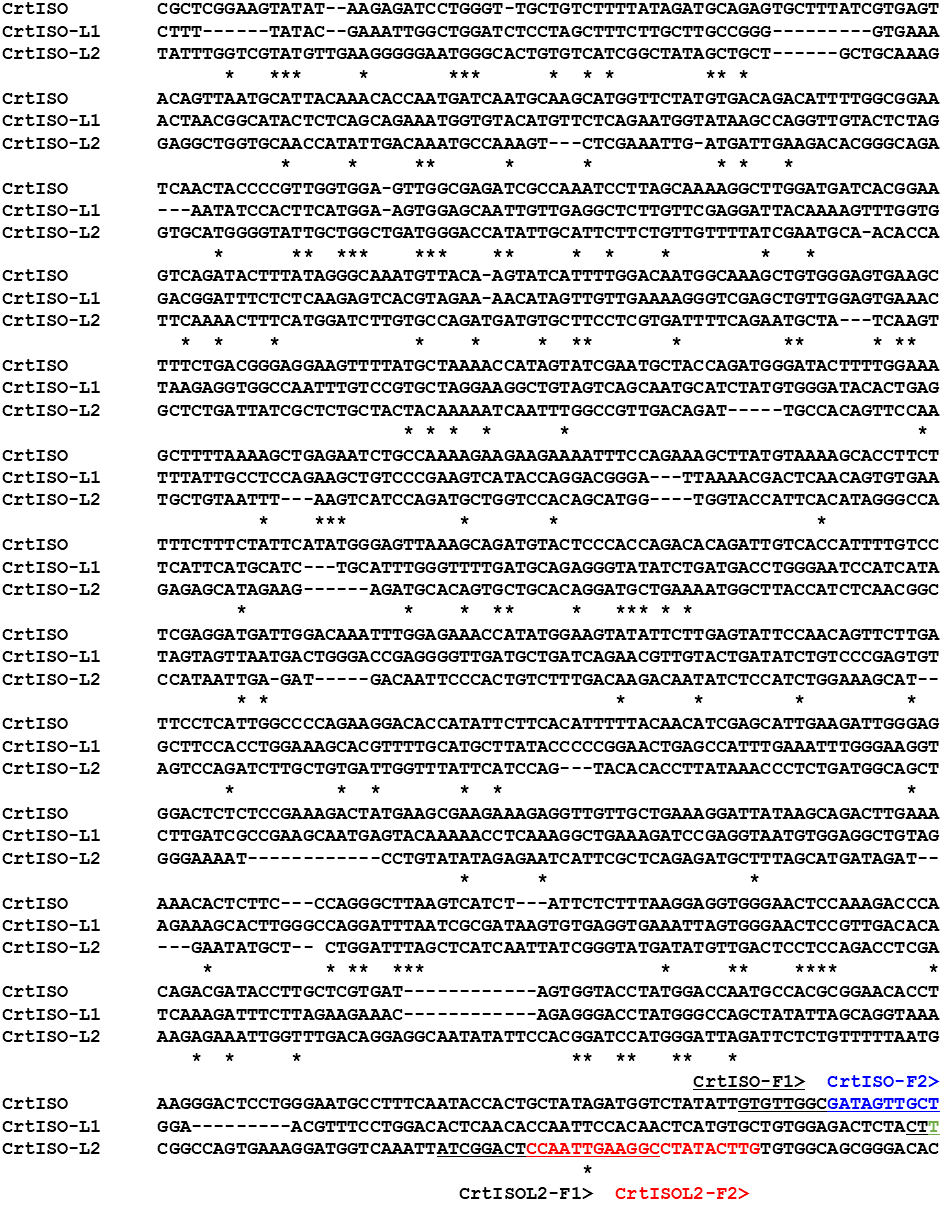


**Figure S7. (Cont.)**


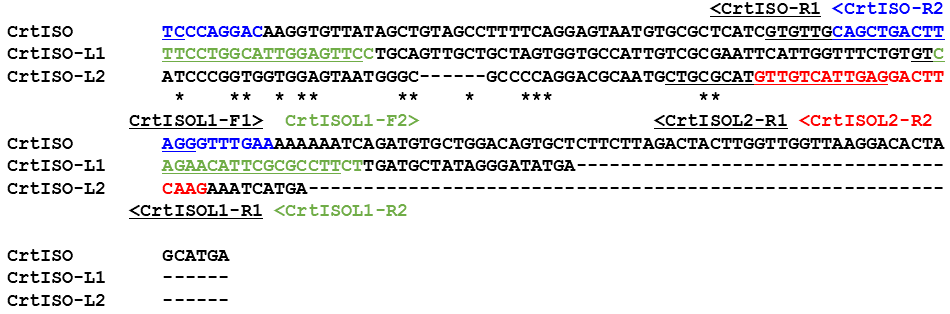


**Figure S8.** **Alignment of the** **cDNA sequences of the tomato *β-LYC1*, *β-LYC2, ε-LCY* and *NSY* genes for qPCR primer design.**


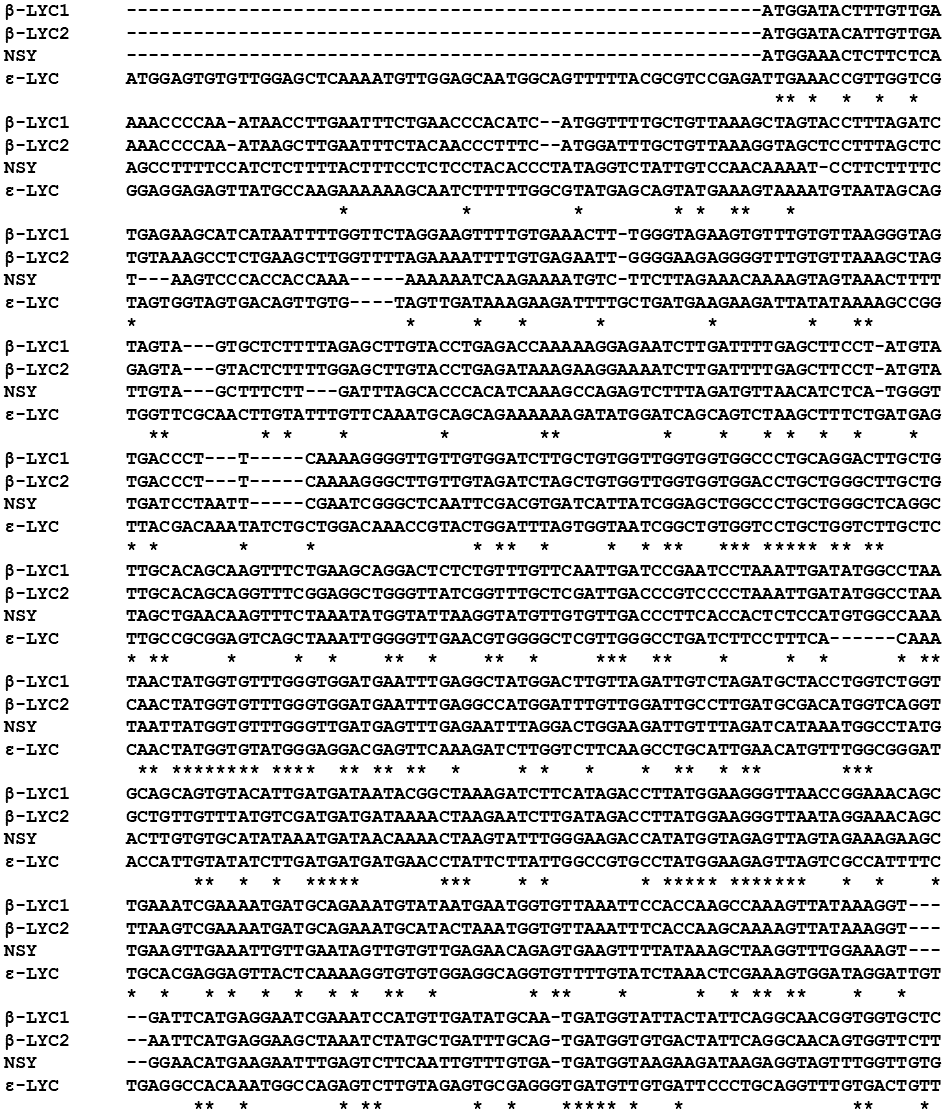


**Figure S8. (Cont.)**

**
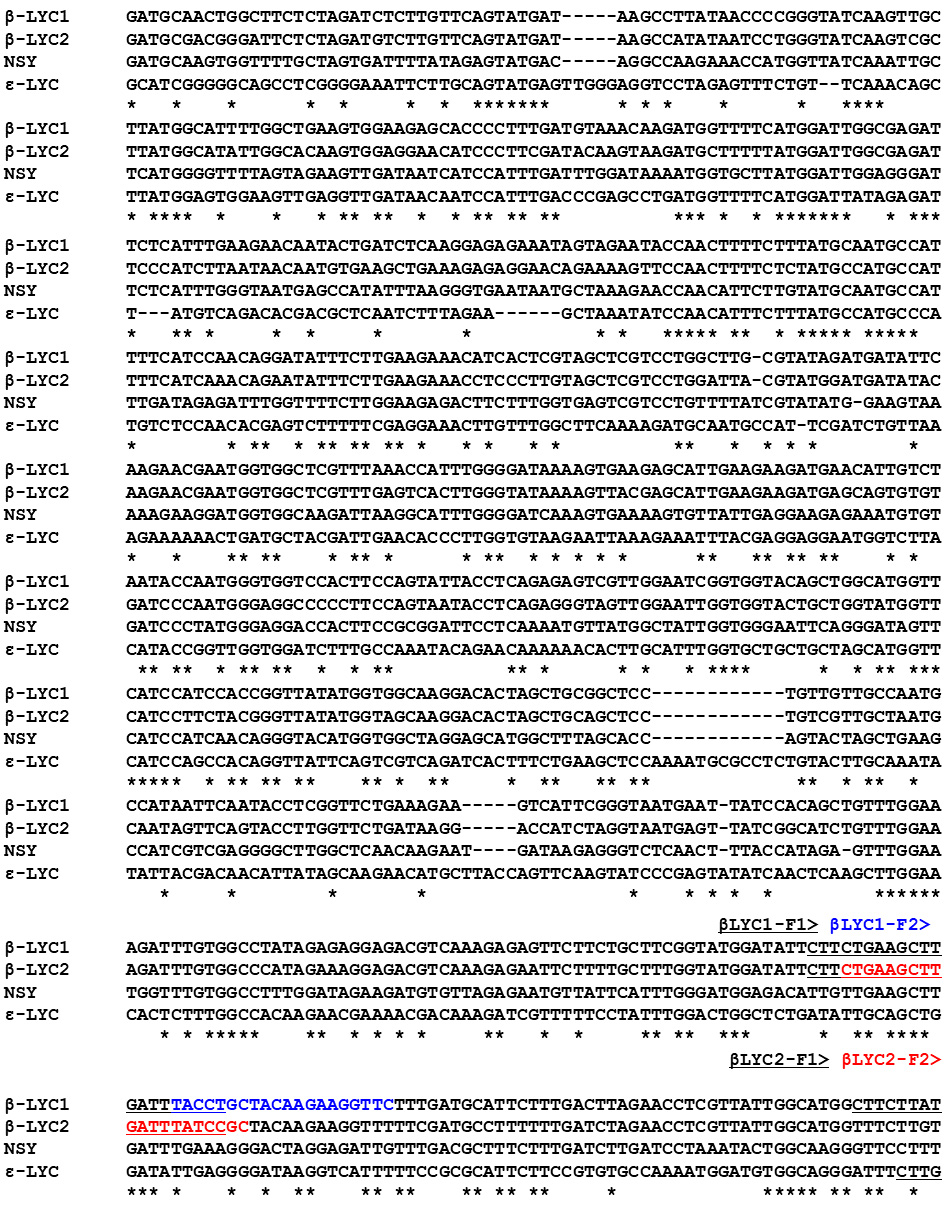
**

**Figure S8. (Cont.)**

**
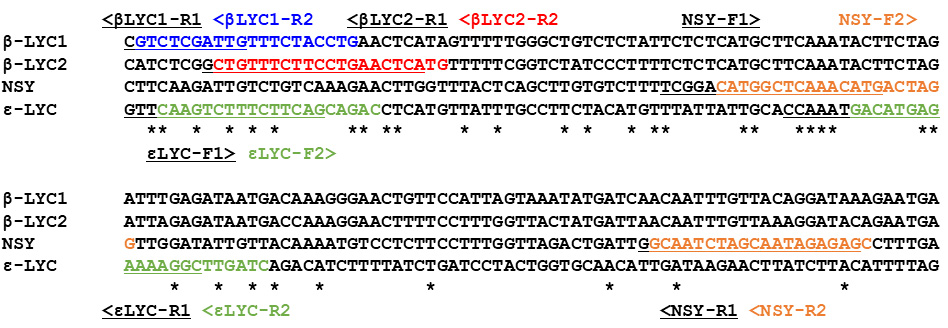
**

**Figure S9.** **Alignment of the** **cDNA sequences of the tomato *CYP97A29* and *CYP97C11* genes for qPCR primer design.**


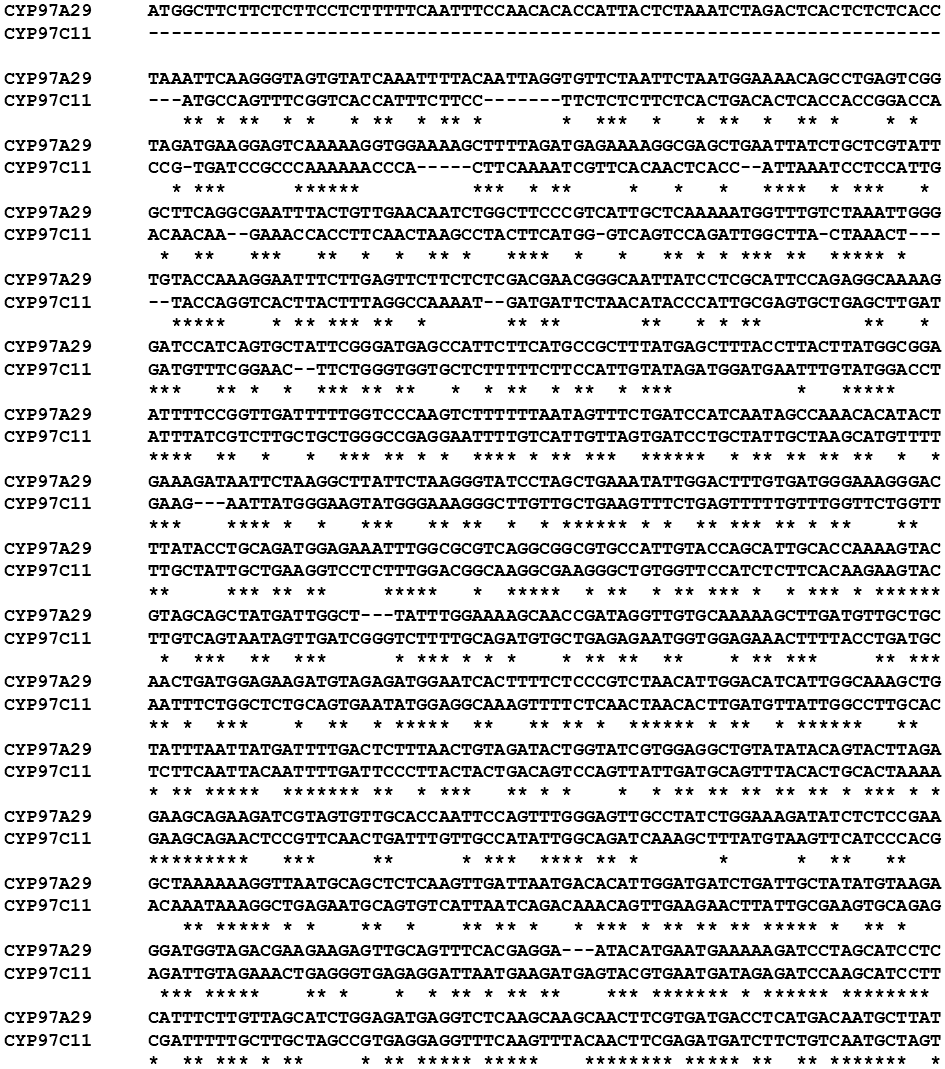


**Figure S9. (Cont.)**

**
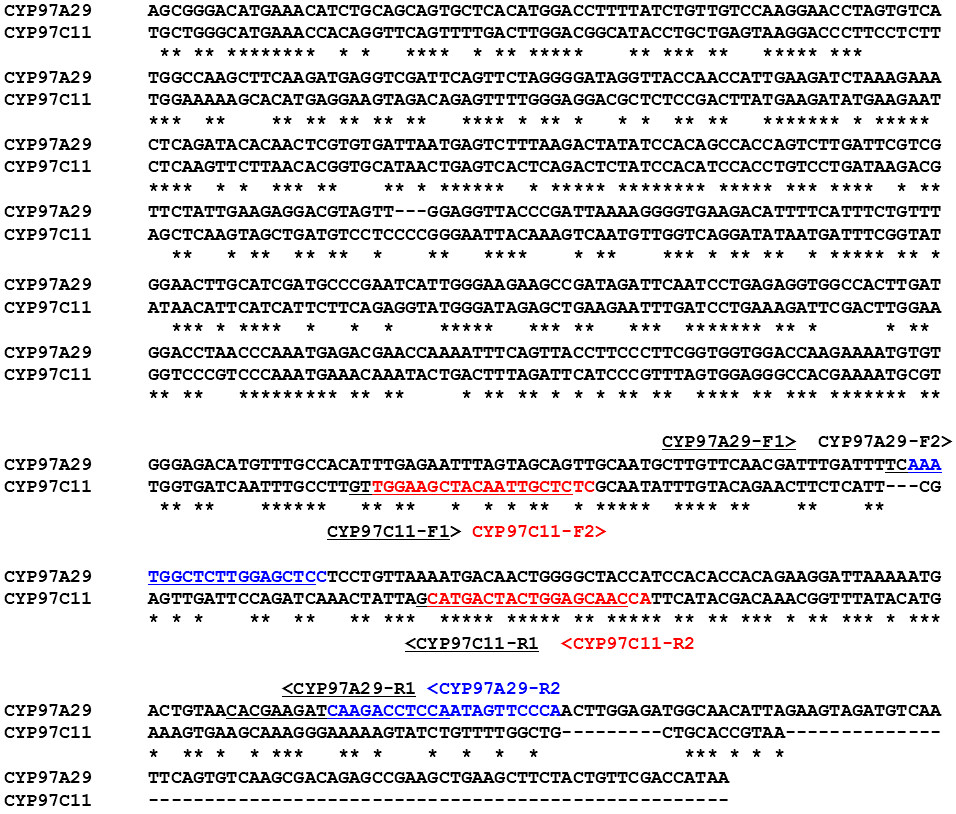
**

**Figure S10.** **Alignment of the** **cDNA sequences of the tomato *BCH1* and *BCH2* genes for qPCR primer design.**


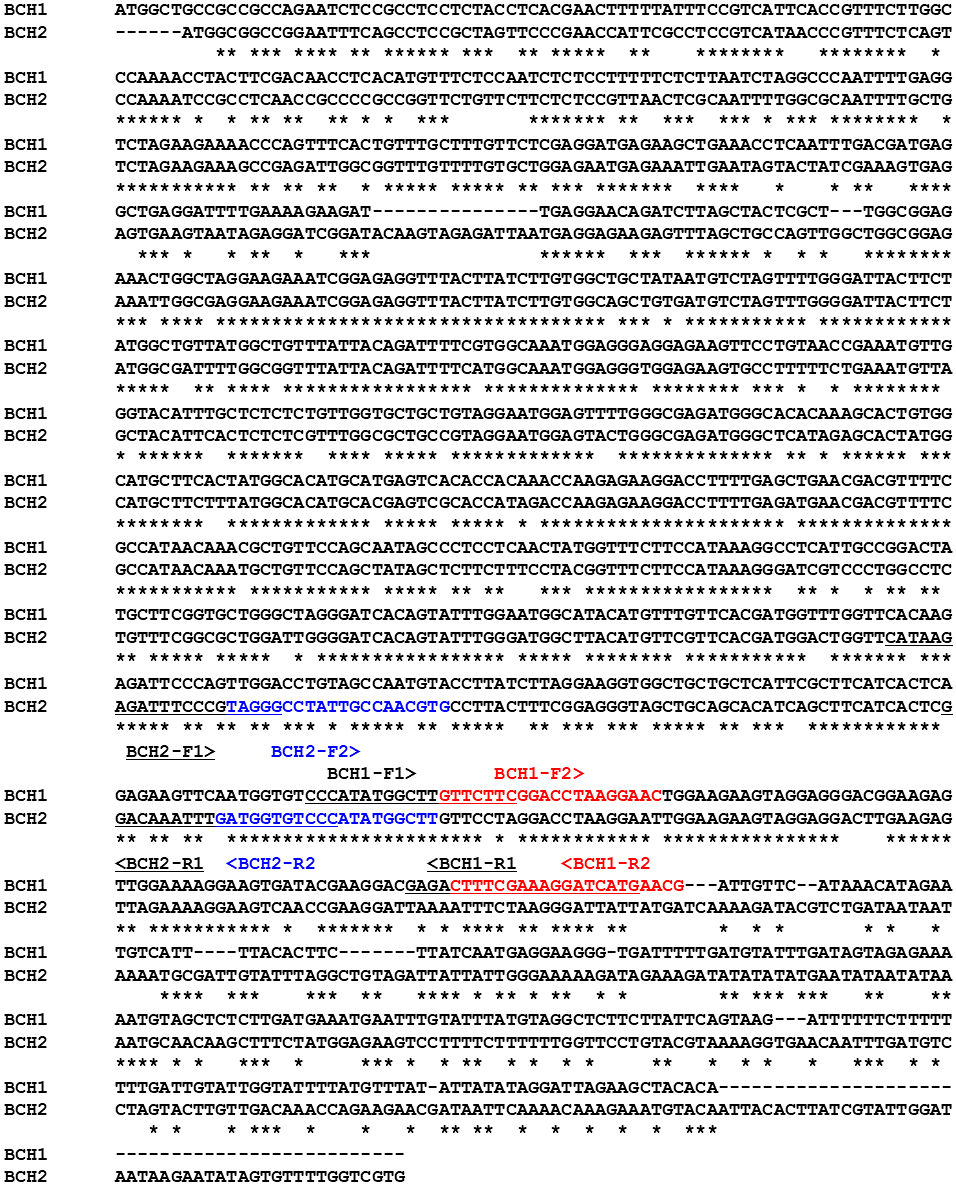


**Figure S11.** **The** **cDNA sequence of the tomato *ZEP* gene for qPCR primer design.**


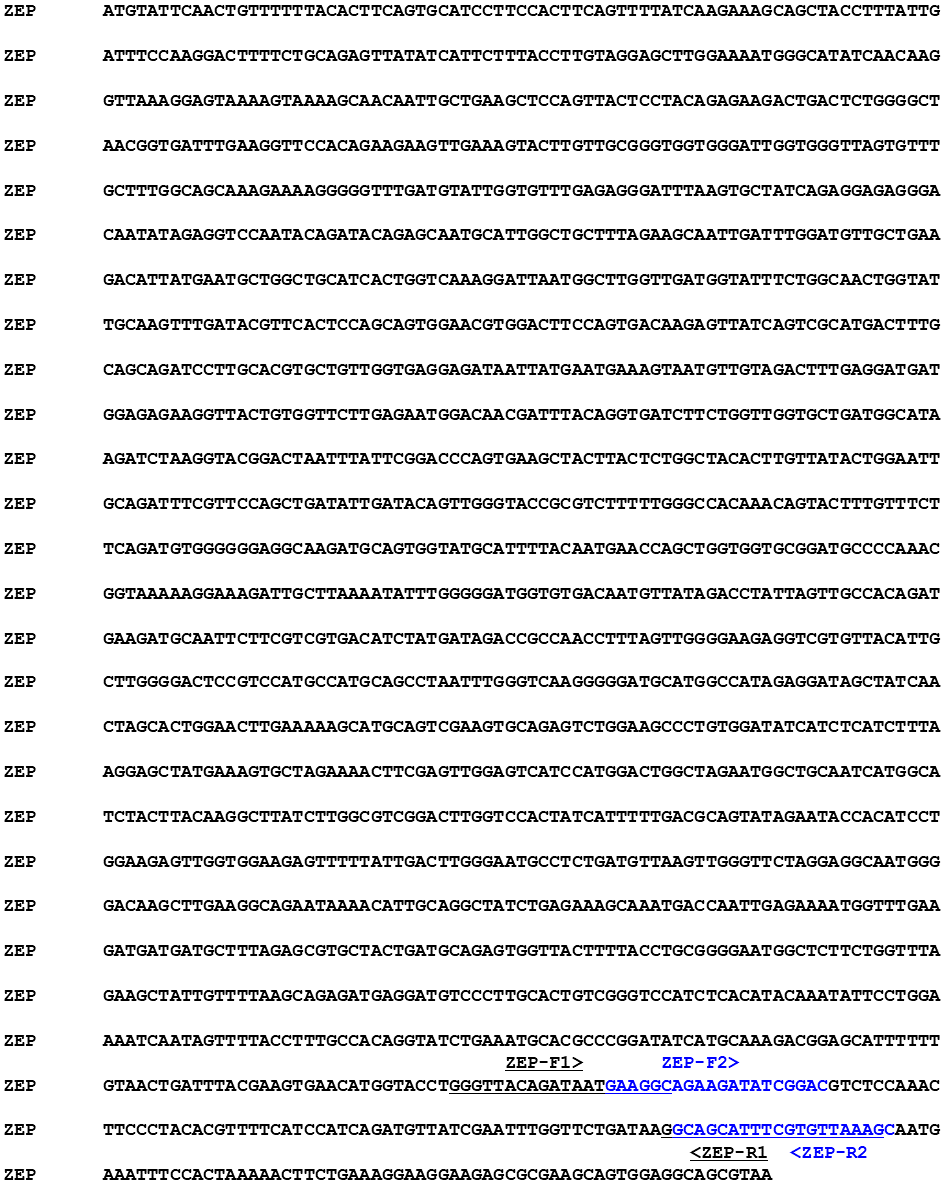


**Figure S12.** **The** **cDNA sequence of the tomato *VDE* gene for qPCR primer design.**

**
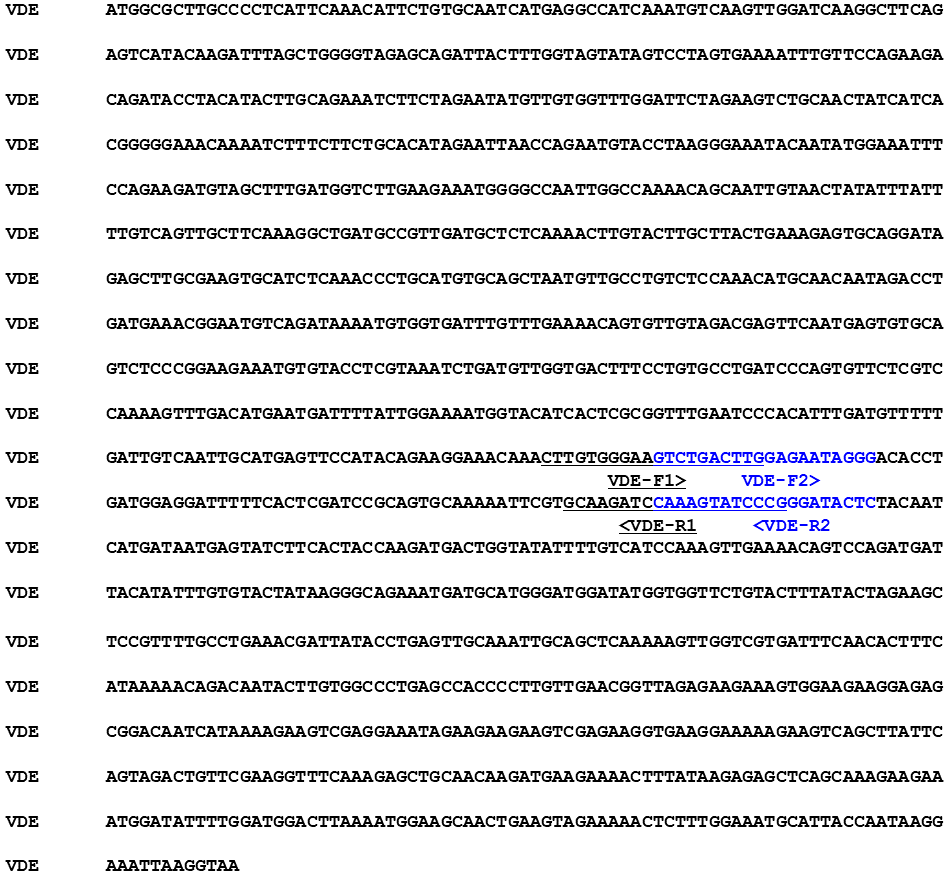
**

**Figure S13. The relative expression levels of the upstream genes in fruits of the 42 potential HLYs at the breaker and red ripe stages measured by qPCR.** The relative expression of each gene was measured by qPCR, and relative quantification was performed using the optimized method^49^ with the tomato *Expressed* and *CAC* genes as the reference genes. The left and right columns of each gene represent the relative expression levels of that gene at the breaker and red ripe stages, respectively. Orange, the genes had significantly higher relative expression levels in individual HLYs at each stage than that in Moneymaker at that stage. Green, the genes had significantly lower relative expression levels in individual HLYs at each stage than that in Moneymaker at that stage. Grey, the genes had insignificantly different relative expression levels in the 42 HLYs at each stage as a group when compared to the non-functional *crtiso* in NC 1Y. Light blue, the genes had significantly higher relative expression levels in individual HLYs at each stage than that in the wild tomato at that stage. Blue, the genes had significantly lower relative expression levels in individual HLYs at each stage than that in the wild tomato at that stage. Pink, the genes had significantly lower relative expression levels at the red ripe stage than at the breaker stage of the same genotypes. Red, the genes had significantly higher relative expression levels at the red ripe stage than at the breaker stage of the same genotypes. Statistics were conducted using two-tailed student’s t-test with two-sample unequal variance, i.e., significantly different expression from that in controls.


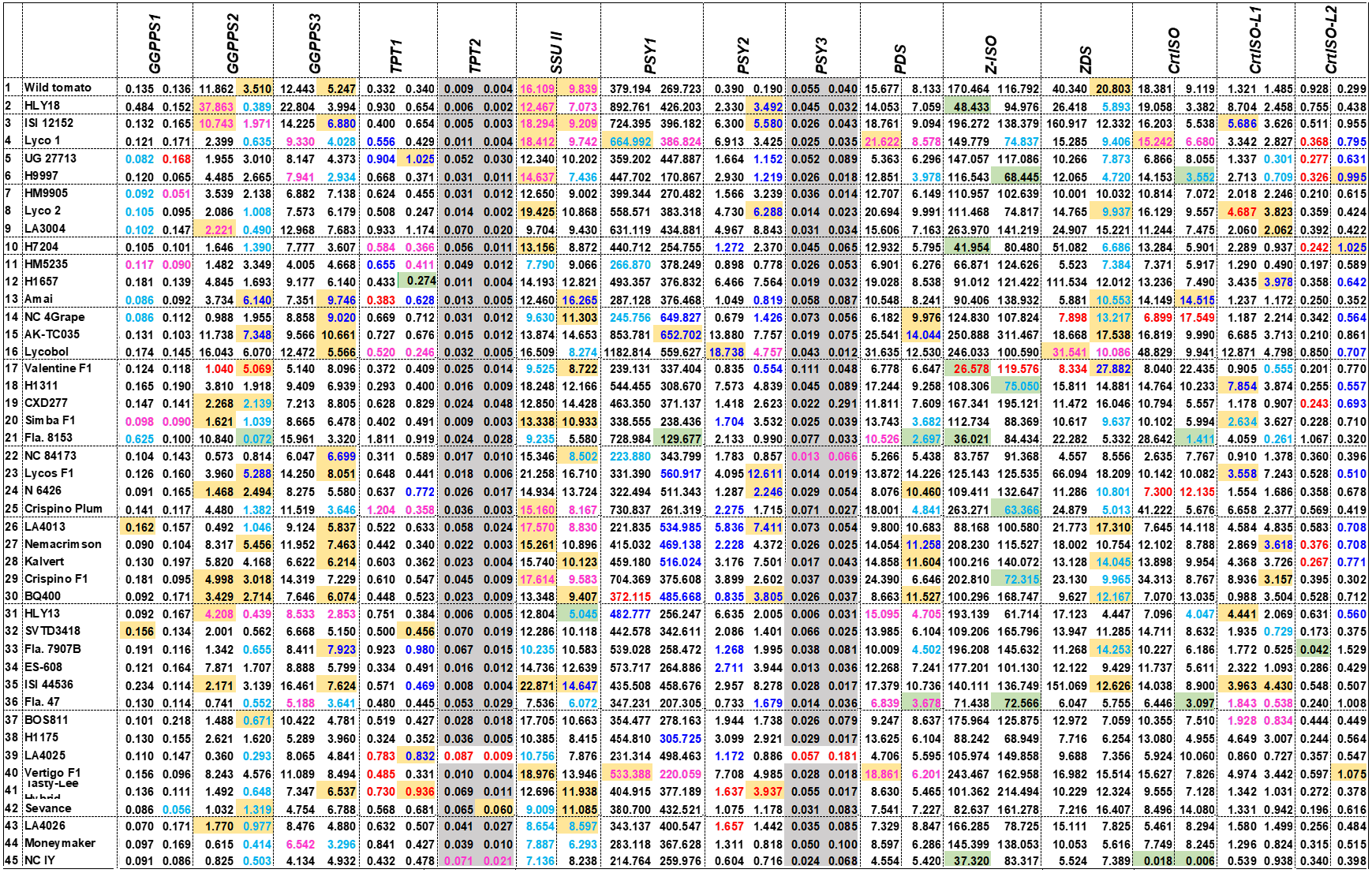


**Figure S14. The relative expression levels of the downstream genes in the fruits of the 42 potential high-lycopene genotypes at the breaker and red ripe stages measured by qPCR.** The relative expression of each gene was measured by qPCR, and relative quantification was performed using optimized method^49^ with the tomato *Expressed* and *CAC* genes as the reference genes. The left and right columns of each gene represent the relative expression levels of that gene at the breaker and red ripe stages, respectively. Orange, the genes had significantly higher relative expression levels in individual HLYs at each stage than that in Moneymaker at that stage. Green, the genes had significantly lower relative expression levels in individual HLYs at each stage than that in Moneymaker at that stage. Grey, the genes had insignificantly different relative expression levels in the 42 HLYs at each stage as a group when compared to the non-functional *crtiso* in NC 1Y. Light blue, the genes had significantly higher relative expression levels in individual HLYs at each stage than that in the wild tomato at that stage. Blue, the genes had significantly lower relative expression levels in individual HLYs at each stage than that in the wild tomato at that stage. Pink, the genes had significantly lower relative expression levels at the red ripe stage than at the breaker stage of the same genotypes. Red, the genes had significantly higher relative expression levels at the red ripe stage than at the breaker stage of the same genotypes. Statistics were conducted using two-tailed student’s t-test with two-sample unequal variance, i.e., significantly different expression from that in controls.

**
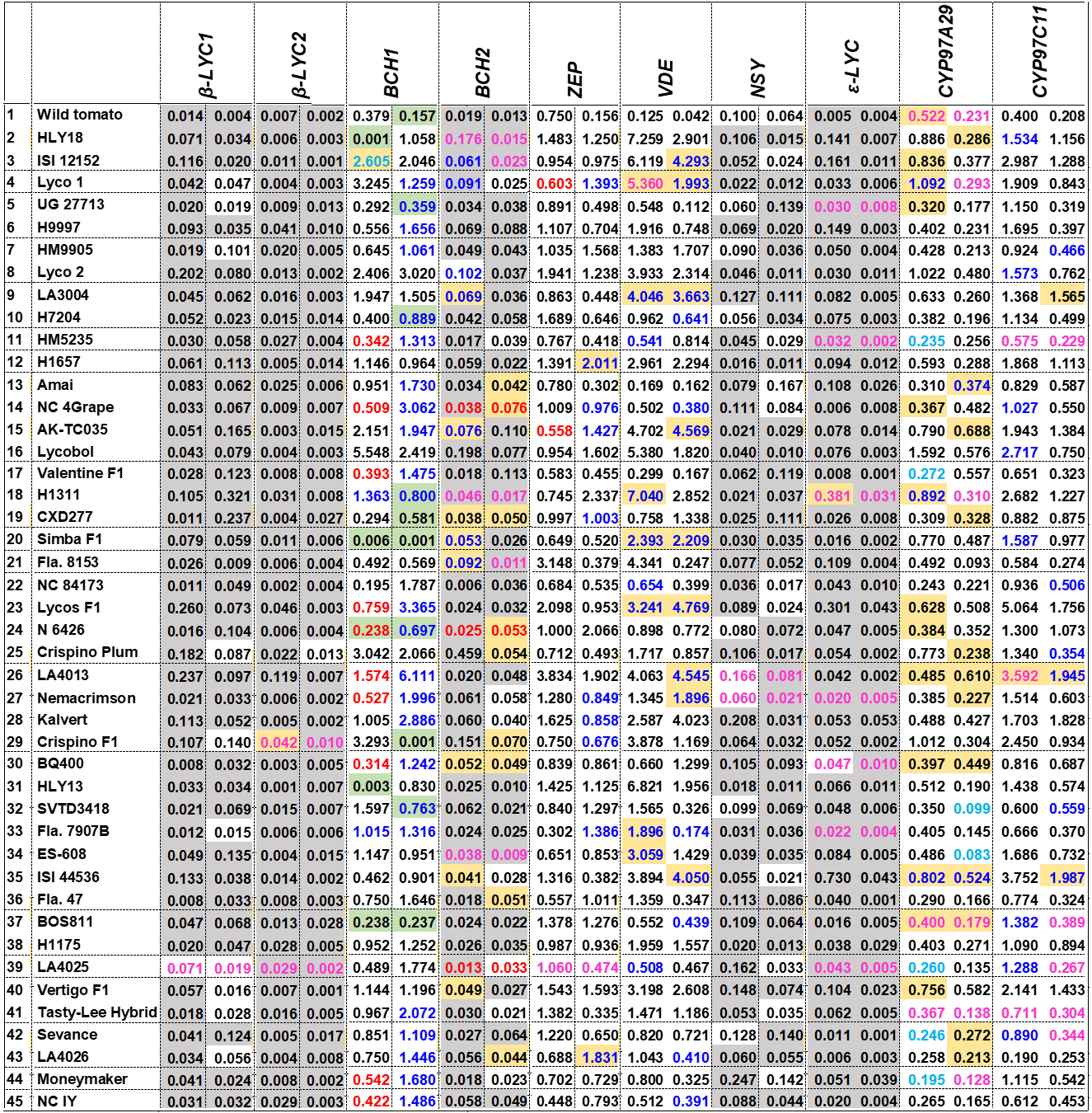
**
